# Conformational Profile of Galactose‐α‐1,3‐Galactose (α‐Gal) and Structural Basis of Its Immunological Response

**DOI:** 10.1002/chem.202500050

**Published:** 2025-03-18

**Authors:** Golokesh Santra, Dimitrios A. Pantazis

**Affiliations:** ^1^ Max-Planck-Institut für Kohlenforschung Kaiser-Wilhelm-Platz 1 45470 Mülheim an der Ruhr Germany

**Keywords:** disaccharides, conformational analysis, molecular dynamics, alpha-gal syndrome, anaphylaxis

## Abstract

Small carbohydrates present a rich and complex conformational landscape whose accurate description is a significant challenge for computational molecular science, yet essential for understanding their physicochemical properties, biological roles, and medical implications. Galactose‐α‐1,3‐galactose (α‐Gal) is a notable example of a disaccharide that remains insufficiently characterized despite being implicated in the life‐threatening anaphylactic response known as alpha‐Gal syndrome. Here we present a thorough conformational analysis of α‐Gal using a unique combination of techniques, ranging from classical dynamics to a staged automatic conformer generation and screening using a quantum‐mechanics‐based protocol elaborated in the present work. The results reveal a remarkably constrained and rigid conformational profile that is minimally responsive to solvation. Subsequently, we study the binding of α‐Gal to the M86 antibody using multiscale hybrid (QM/MM) calculations. Quantum mechanical analysis of the binding in terms of non‐covalent interactions, local energy decomposition, and quantities derived from the quantum theory of atoms in molecules, enable us to identify and quantify the key interactions that form the structural basis of α‐Gal's immunological response.

## Graphical abstract

A multilevel computational analysis of the disaccharide α‐Gal describes in detail its rigid conformational profile in solution, while multiscale hybrid (QM/MM) calculations, energy decomposition and analysis of non‐covalent interactions are used to probe the atomistic details of the binding of the disaccharide to the M86 antibody.

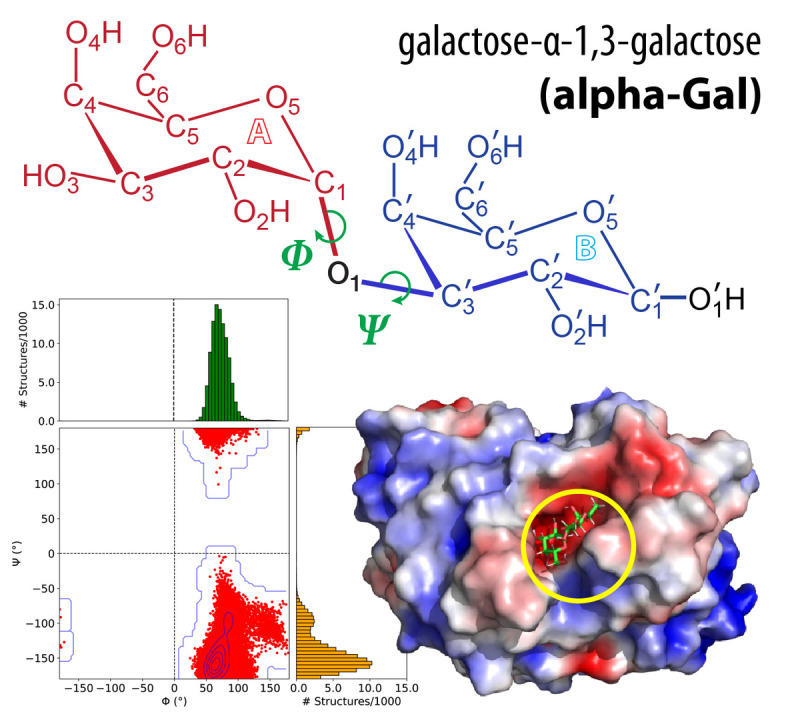

## Introduction

Disaccharides, carbohydrates generated by linking diverse monosaccharides through a single glycosidic bond, are a major class of biomolecule with fundamental significance for life. As with their longer‐chain congeners, their properties and biological roles are intimately related to their conformational profiles, which are unique for each monosaccharide combination and type of glycosidic linkage. These profiles are controlled by the glycosidic bond, the conformation of each constituent ring, and the multiple intra‐ and inter‐molecular hydrogen‐bonding possibilities involving the hydroxyl groups.^[1]^ Atomic‐level understanding of these conformational profiles is essential for elucidating chemical properties and deciphering structure‐dependent biological functions of the disaccharides themselves as well as of related oligosaccharides. Most naturally occurring disaccharides are typically associated with nutrition, but in certain cases they can have adverse health effects. These are either connected to specific intolerances, such as in the case of lactose, or more rarely to severe allergic reactions (anaphylaxis). The latter is the case for galactose‐α‐1,3‐galactose, commonly known as α‐Gal, which is the subject of the present work.

**Figure 1:**
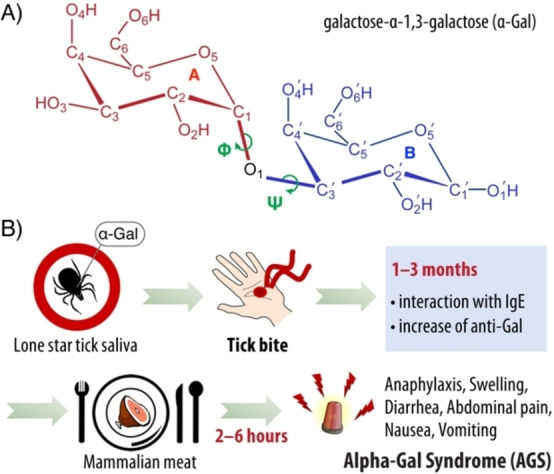
A) Chemical structure of galactose‐α‐1,3‐galactose. The two rings are shown in red and blue color and labelled as A and B, respectively. The α‐1,3 glycosidic linkage is formed along $C_{1}$ −$O_{1}$ −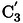 , whereas $∠O_{5}$ −$C_{1}$ −$O_{1}$ −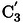 and 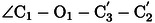 are the dihedral angles Φ and Ψ, respectively. B) Schematic representation of the alpha‐gal syndrome (AGS) originating from the lone star tick bite.

Exposure to α‐Gal produces an unusually large amount of anti‐Gal IgE, which is responsible for the tick bite induced mammalian meat allergy known as alpha‐gal syndrome (see Figure 1), cetuximab‐induced anaphylaxis, a possible risk factor for coronary artery disease, and rejection of pig‐to‐primate xenotransplantation.[^2^, ^3^, ^4^, ^5^, ^6^, ^7^] The natural antibody, anti‐Gal, is present in very high concentration (~1 %) in human serum.[^8^, ^9^, ^10^, ^11^] It exhibits a distinct specificity for α‐Gal, a terminal moiety of the Galα1‐3Galβ1‐4GlcNAc‐R (α‐galactosyl) epitope, abundantly expressed in cells of non‐primate mammals, prosimians, and New World monkeys. In these organisms, α‐galactosyl is produced by α‐1,3‐galactosyltransferase (α1,3GT), which is absent in humans, apes and Old World monkeys.[^12^, ^13^] Yilmaz *et al*. have argued that protection against malaria may have caused the exclusion of the gene responsible for α1,3GT activity during human evolution.^[14]^ The structural basis for recognition and binding of α‐Gal to antibody was elusive until Langley *et al*. solved the crystal structure of α‐Gal in complex with the M86 and HKB7 antibodies.^[15]^ The crystallographic models offered a first broad view into structural factors that control binding,^[16]^ but a precise and quantitative characterization of specific interactions is highly desirable.

Remarkably, despite α‐Gal's importance, a complete characterization of even its intrinsic conformational profile is still lacking. Due to extensive possibilities for hydrogen bonding and the inherent conformational flexibility of the galactose rings, α‐Gal's conformational space is expected to be intricate and diverse. It is well known that experimental approaches like X‐ray crystallography and NMR cannot capture the complete range of accessible conformations that may be relevant for understanding molecular behavior within a solvent or biological environment. Therefore, computational studies are essential for exploring the conformational space, which grows exponentially with the number of “rotatable” bonds. Sampling techniques include various types of molecular dynamics (MD) simulations,[^17^, ^18^, ^19^] “basin‐hopping” global optimizations,[^20^, ^21^] rule‐based conformer generators,[^22^, ^23^, ^24^, ^25^, ^26^] and tools such as Grimme's metadynamics‐based algorithm, the Conformer–Rotamer Ensemble Sampling Tool (CREST).[^27^, ^28^] Historically, conformational studies of disaccharides have relied on molecular mechanics methods,[^29^, ^30^, ^31^, ^32^, ^33^, ^34^] but more recently a shift toward combining conformer generation tools with density functional theory (DFT) optimizations is advocated for reliably mapping conformation to chemical reactivity and the variable electronic structures.[^35^, ^36^, ^37^, ^38^, ^39^] For several organic compounds, CREST conformer generation followed by DFT optimization has been suggested as a reliable tool for calculation of conformer energies and other properties.[^38^, ^39^, ^40^]

The present study leverages these developments to address the open questions regarding both the intrinsic conformational properties of α‐Gal and its binding with the antibody. The first objective is the accurate sampling of the conformational space of α‐Gal in water, employing both force‐field based MD simulations and a conformer generation and evaluation approach, where conformers generated with the CREST algorithm at the semiempirical extended tight‐binding (xTB) level are refined using DFT optimizations to accurately map the conformational landscape of α‐Gal and reliably identify global as well as local minima. The second objective of the present work is the characterization of specific interactions by hybrid quantum mechanics/molecular mechanics (QM/MM) modelling of α‐Gal binding to the M86 antibody. This is coupled to a host of quantum chemical analyses that lead to precise identification and quantification of specific strong and weak interactions. Overall, the present work offers novel insights both into the intrinsic conformational landscape of α‐Gal and on the molecular basis of its immunological response in terms of disaccharide–antibody recognition and binding.

## Methodology

### Molecular Dynamics Simulations

MD simulations were performed using AMBER with the GLYCAM06 force field,[^41^, ^42^] and the OPC water model.[^43^, ^44^] A truncated octahedron box with 1042 water molecules was used for solvating α‐Gal. Hydrogens were constrained with SHAKE.[^46^, ^47^] NPT simulations were carried out at a temperature of 300 K and a pressure of 1 atm. The Langevin thermostat and Monte Carlo barostat were used for temperature and pressure control.[^48^, ^49^] The non‐bonded cutoff was set to 8.0 Å. A total duration of 1 μs was used for the production MD simulation (2 fs time step) running on a single GPU. 100 000 snapshots were extracted for further analysis.^[45]^

### CREST and DFT Calculations

CREST 3.0 was used to explore the conformers/rotamers of α‐Gal,[^27^, ^28^] employing the semi‐empirical tight‐binding method GFN2‐xTB^[50]^ and the analytical linearized Poisson‐Boltzmann (ALPB) model for implicit water solvation.^[51]^ A 0–6 kcal/mol relative energy range was employed for conformer generation. Subsequently, geometry optimizations were performed using the DFT functionals r^2^SCAN‐3c,^[52]^ r^2^SCAN‐D4^[53]^ and r^2^SCAN0‐D4.^[54]^ The SMD implicit solvation model^[55]^ was used in DFT calculations. All electronic structure calculations were performed using ORCA 6.0.^[56]^ Optimizations used the DefGrid3 integration grid, TightOpt convergence, and the RIJCOSX approximation,^[57]^ with the def2‐TZVPP basis sets and corresponding def2/J auxiliary basis sets for the r^2^SCAN‐D4 and r^2^SCAN0‐D4 calculations.^[58]^

### Studies of the α‐Gal‐Antibody Complex

All‐atom models were created after careful preparation of the original structure with PDB ID 7UEN. The completed structure along with crystallographic waters were solvated in a box with 15 Å distance between its edges and the protein. Simulations were performed under periodic boundary conditions using the FF19SB force field for the protein,^[59]^ the TIP3P water model,^[60]^ and GAFF2 for α‐Gal. After solvent minimization, a short NPT simulation was carried out with restraints on the protein and ligand complex and a frame was selected for subsequent QM/MM calculations. Three QM/MM models with different QM and active regions (i.e. regions fully relaxed in either QM or MM layers) were considered: two relatively small models (**SM1** and **SM2**, with QM regions of 156 and 182 atoms, respectively, and a total active region of 707 atoms for both) and a large model (**LM**, with 250 QM atoms and a total of 1095 active atoms in both QM and MM). Detailed definition and description of the models is provided in the SI. All models explicitly incorporate the residues identified as critical for CH‐π interactions and hydrogen bonds between α‐Gal and the M86 antibody.^[15]^ QM/MM optimizations were performed using the BP86 functional,^[61]^ def2‐TZVP basis set,^[58]^ and def2/J auxiliary basis set. Single‐point calculations were carried with the B3LYP functional,[^62^, ^63^] and increased integration grids. Calculations with the domain‐based local pair natural orbital coupled‐cluster method with single, double, and perturbative triple excitations, DLPNO‐CCSD(T),[^64^, ^65^, ^66^, ^67^, ^68^] utilizing the def2‐TZVP basis sets^[58]^ and TightPNO thresholds, were performed as basis for the local energy decomposition (LED) analysis.^[69]^ All QM/MM calculations were performed using ORCA.^[56]^ Further analysis was conducted using Multiwfn^[70]^ and NCIPLOT4.^[71]^

## Results and Discussion

### Molecular Dynamics Simulations

The first target is to establish the conformational profile of α‐Gal in solution using complementary methodologies. The conformational flexibility of disaccharides is predominantly determined by the existence of different rotamers around the glycosidic linkage. Although the six‐membered rings exhibit a certain degree of flexibility themselves, this is somewhat constrained in stable oligo‐ and polysaccharides, therefore conformational studies mainly focus on the glycosidic torsional angles Φ and Ψ. For definitions specifically on α‐Gal, see Figure 1.

**Figure 2:**
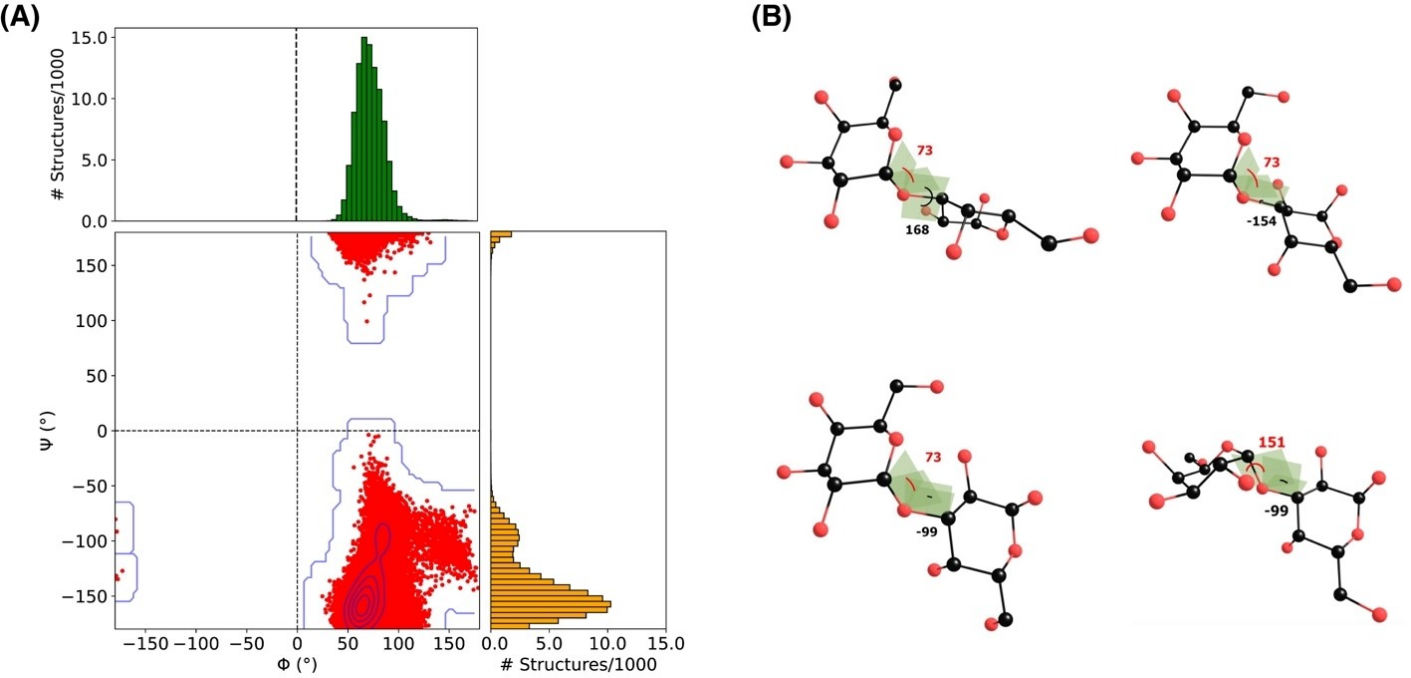
A) Φ vs. Ψ scatter plot of α‐Gal extracted from 100 000 MD simulation frames. The distribution of each dihedral angle is depicted using histograms in panels parallel to the corresponding axis. B) Depiction of four α‐Gal structures from representative regions of the plot.

As a first approach, we employ force‐field based MD simulations, which have been widely used to study the conformational space of oligosaccharides in explicit solvent.[^19^, ^42^, ^72^, ^73^, ^74^, ^75^, ^76^] Analyzing the Φ vs. Ψ plot from our 1 μs trajectory (Figure 2), it is evident that MD predicts α‐Gal to have a clustered distribution in terms of the Φ dihedral, mainly in the positive Φ region with a maximum close to 73°. Ψ angles are clustered within the negative region, with a maximum around ‐150°. A secondary maximum in the Ψ distribution is observed around ‐90°; around this Ψ region there is also a small spread of conformations with Φ centered around 150°, however the overall population of this sub‐cluster is small (Figure 2). We note that positive Ψ values (>150°) correspond to the continuation of the major cluster, i.e. conformers where the B ring has turned from negative to positive dihedral values. The above observations differ from a previously reported MM3 simulated potential energy surface of α‐Gal, which showed a similar narrow distribution of Φ but three distinct clusters in the Ψ dihedral.^[77]^ Average values of Φ and Ψ from the present MD simulations are 73.4±14.3° and −143±27.7°, respectively.

Following previous works[^1^, ^19^, ^33^, ^34^] in assuming that a distance shorter than 2.5 Å between H and an acceptor is indicative of an intramolecular hydrogen‐bond (HB), analysis of the MD trajectory shows that 72 % of the α‐Gal snapshots have no intramolecular HB, while 26 % have only one intramolecular HB (Table S1). Among these 26148 snapshots with a single HB, 1982 have the O_6_H⋅⋅⋅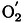 hydrogen bond. This bond is important because it is also observed when α‐Gal binds to the M86 antibody (see below). Although MD simulations at physiological conditions (NPT) are advantageous to capture the dynamical behavior of a disaccharide in explicit solvation, the inter‐ and intra‐molecular interactions reflect the limitations of the force field used to describe chemical bonding and weak interactions. That is why it is essential to complement such studies with quantum mechanical approaches.

### Automated Conformer Generation and QM‐Based Ranking

**Figure 3:**
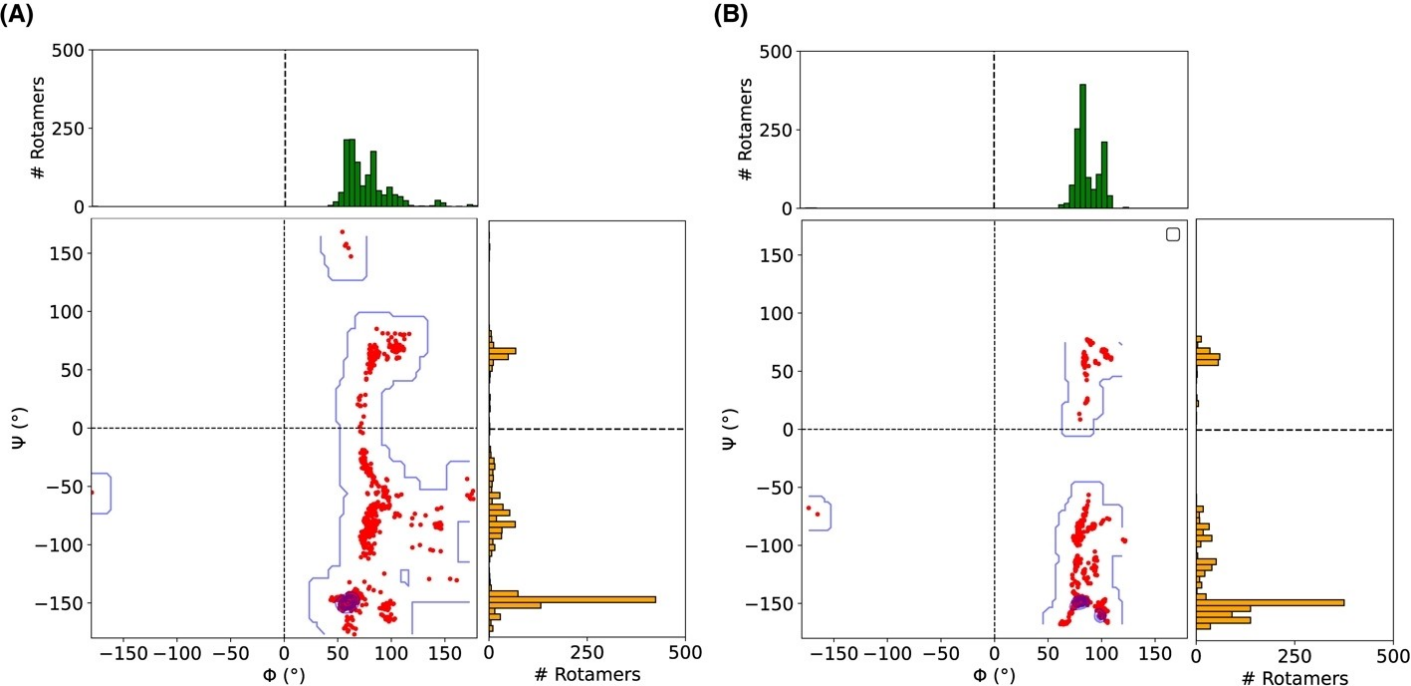
The Φ vs. Ψ scatter plot of 1270 rotamers, A) generated from CREST and B) reoptimized using r^2^SCAN0‐D4. Each dihedral angle distribution is depicted using histograms in panels parallel to the corresponding axis.

Here we present an alternative protocol that relies on a staged approach, where the automated generation of a conformer–rotamer ensemble using the CREST algorithm based on a semi‐empirical method is followed by successive DFT‐based screening and evaluation. 1270 unique conformers/rotamers of α‐Gal were generated using CREST with the semi‐empirical tight‐binding method GFN2‐xTB^[50]^ and the analytical linearized Poisson‐Boltzmann (ALPB) implicit solvation model for water.^[51]^ The Φ dihedrals of the CREST‐generated rotamers are more localized than the Ψ dihedral angles, with the majority of structures concentrated within the Φ=50°–150° range. In contrast to the MD simulation results, the distinct clustering nature of the Ψ dihedrals is absent in the CREST‐generated structures (Figure 3A). The much broader scatter suggests a distinct underlying description of relative conformer energetics between the two approaches. Considering the GFN2‐xTB energies, the Φ and Ψ angles of the lowest energy rotamer are 65.1° and −145.7°, respectively. The glycosidic angle of this rotamer is 115°. The lowest energy conformer at the GFN2‐xTB level has two intramolecular hydrogen bonds. Among the 1270 structures, 42 % have one, and another 42 % have two intramolecular HBs (Figure S1).

Taking one step further, all rotamers generated by CREST were optimized using a dispersion‐corrected modern meta‐GGA functional, r^2^SCAN‐D4^[53]^ and solvation model based on the density (SMD).^[55]^ All DFT calculations were performed with the ORCA package.^[78]^ The outcome of this step is that the Φ and Ψ distribution become more narrow (Figure S2). Refining further the results with the higher‐level hybrid functional r2SCAN0‐D4,^[54]^ we observed no additional significant changes in the distribution pattern of the Φ and Ψ dihedrals (Figure 3B). The distribution of Ψ dihedrals is largely shared between two regions. Transitioning from one region to another can be achieved simply by rotating the B‐ring along the second glycosidic bond (i. e., 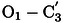 ). The conformers with large positive Ψ and Φ angles (≥130°) were present among the MD snapshots but are no longer present in the CREST/r^2^SCAN0‐D4 ensemble. 17 % of the structures have no intramolecular HBs, whereas 40 % have only one (Table S1). Among the 504 rotamers with a single HB, only three have the O_6_H⋅⋅⋅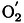 hydrogen bond.

**Figure 4:**
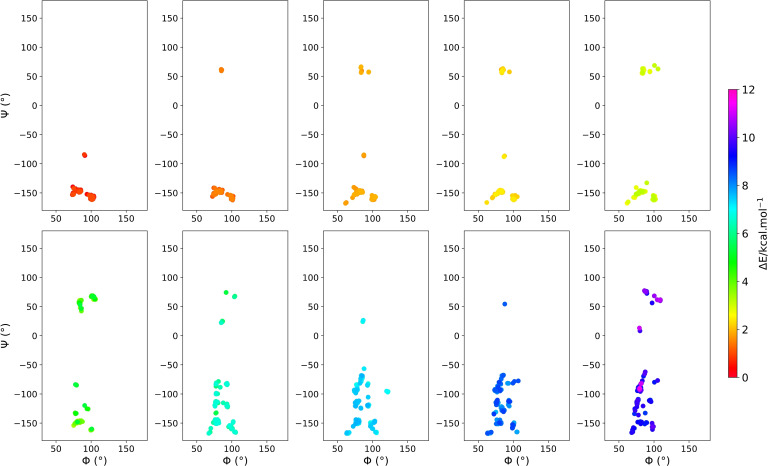
Depiction of energy‐screened population of Φ vs. Ψ graphs according to the r^2^SCAN0‐D4 relative energies for the 1270 optimized rotamers, with respect to the global minimum. Each of the ten panels contains successively 127 rotamers.

After ranking the structures based on the highest‐level r^2^SCAN0‐D4 energies, we found that the most stable structure has Φ=102.8°, Ψ=−160.0°, three intramolecular HBs, a 115.9° glycosidic angle, and glycosidic bond distances of 1.41 Å and 1.43 Å. Among 1270 structures, only about 15 % reside in the positive Ψ region. The lowest‐energy structure from that region is the 200^th^ rotamer (Φ=85.7°, Ψ=61.1°), which is 1.33 kcal/mol higher in energy (see Figure 4 and supporting animation). Considering the CREST/r^2^SCAN‐D4 rotamers, the local minimum from the “positive Ψ” island is the 131^st^ structure, and it is 1.28 kcal/mol higher in energy than the global minimum (Figure S3b and supporting animation).

Semiempirical quantum mechanical methods such as GFN2‐xTB have been reported to underestimate the relative energies of high‐energy conformers,^[50]^ while some studies have suggested that CREST‐generated conformers require refinement using higher‐level DFT methods for accurate energetics and spectroscopic properties.[^38^, ^40^, ^79^] In view of the present results and the non‐negligible differences in energy profiles between the original CREST ensemble and the final DFT results, we also recommend optimizing GFN2‐xTB generated conformers with a more accurate DFT method, such as r^2^SCAN0‐D4, to obtain a reliable final ranking of conformers.

There are still noticeable differences in the distribution of Φ and Ψ angles and in the intramolecular hydrogen bonding in the conformers generated by the two different approaches employed here, i. e. molecular dynamics (MD) and CREST/r^2^SCAN0‐D4. Besides the fundamentally distinct physical basis of the methodologies, a contributing factor could be the solvation treatment. Although it is not technically feasible to evaluate the different solvent representations on a precisely equal footing in terms of electronic structure theory, we can still attempt to assess the effect of implicit solvation by optimizing the CREST‐generated rotamers in the gas phase using the r^2^SCAN−D4 functional. Analysis of the Φ vs. Ψ plot shows that the distribution of the dihedrals differs from that obtained with the SMD solvation model (Figure S2). The lowest‐energy conformer still falls within the “negative Ψ” region, with Φ=89.3°, Ψ=−132.8°, and has four intramolecular HBs. Among the gas‐phase optimized structures, over 75 % have two or more intramolecular hydrogen bonds, dropping to around 45 % with SMD solvation. Without any solvent, only 7 % of structures have zero intramolecular hydrogen bonds (Table S1). The sequence from gas‐phase to continuum solvation to explicit solvation is therefore directly related to the extent of intramolecular hydrogen bonding and in this way, it can affect the final distribution of Φ and Ψ angles. Importantly, however, for the present study, the major qualitative conclusions regarding the conformational preferences and rotamer distribution of α‐Gal remain robust.

In summary, conformational analysis using different methodologies shows that the distribution of Φ is quite narrow and the majority of the structures are clustered on positive values around 50°–60°. The distribution of the Ψ angle is different for the MD and CREST/GFN2‐xTB ensembles, with that of CREST‐generated conformers being much more spread out than the MD. However, after DFT optimization the CREST conformers fall in two clusters. The extent of intramolecular hydrogen bonding depends on the simulation methodology and the treatment of solvation. For example, about 72 % of the α‐Gal structures from MD have no intramolecular hydrogen bonds, compared to only 16 % of the CREST/r^2^SCAN0‐D4 ensemble. These differences highlight the importance of considering multiple independent approaches for evaluating the conformational profiles of such systems. Despite these differences, all methods concur that the Φ distribution is narrower compared to Ψ, and the most populated region of α‐Gal remains largely consistent across all methods based on Φ and Ψ dihedral angles. Following the most refined energy ranking of the present study, the lowest energy state is found in the most populated cluster (with Φ=102.8° and Ψ=−160.0°), and it is 1.33 kcal/mol more stable than the local minimum in the second cluster (with Φ=85.7°, Ψ=61.1°).

### α‐Gal in Complex with the M86 Antibody

To obtain detailed insights into the α‐Gal‐antibody interaction, we utilize the highest resolution crystallographic model (1.55 Å) by Langley *et al*.,^[15]^ which represents α‐Gal complexed with the M86 Fab (PDB ID: 7UEN). After modelling the missing residues with Modeller,^[80]^ the α‐Gal bound protein including the crystallographic waters was solvated in a 15 Å cubic box. Following a short MD equilibration under periodic boundary conditions, one snapshot was chosen to proceed with QM/MM calculations as described in the Supporting Information.

**Figure 5:**
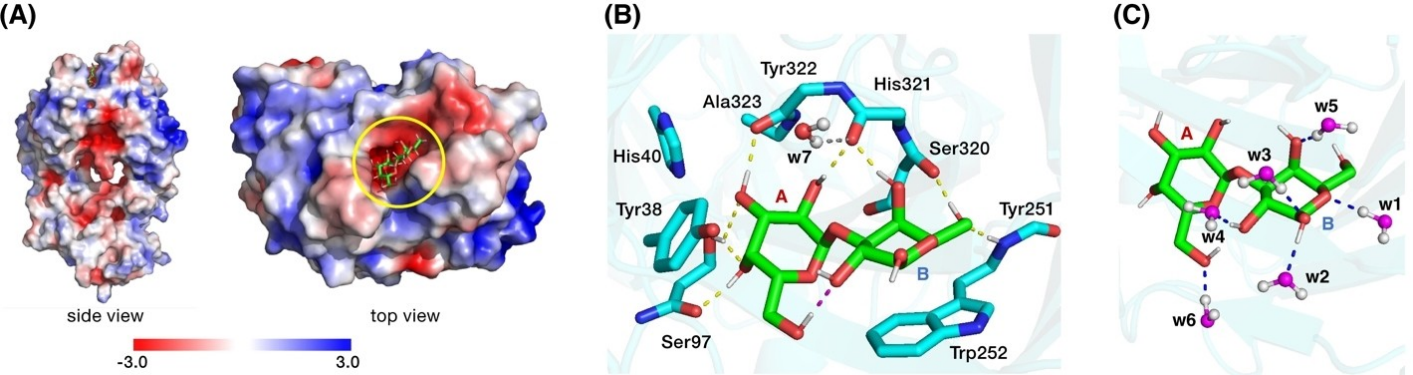
A) Side and top view of the electrostatic potential surface (EPS) of the QM/MM starting structure of α‐Gal‐M86 complex. The protein is colored according to the electrostatic potential, red for negative and blue for positive potential. The ligand α‐Gal is shown as sticks within its binding pocket. B) Optimized QM core of a QM/MM model depicting important surrounding residues of the M86 antibody. Eight hydrogen bonds between α‐Gal and M86, one intramolecular hydrogen bond, and the bond between water w7 and the backbone carbonyl of His321 are highlighted. C) Hydrogen bonding interactions between bound α‐Gal and six bulk waters.

Electrostatic potential surface of the starting structure of our QM/MM models indicates that the α‐Gal binding pocket of M86 presents a negatively charge surface with the A‐ring being more deeply embedded in the protein matrix compared to the B‐ring (Figure 5A). We defined three distinct QM regions for our QM/MM models, labelled SM1 (156 atoms in QM region), SM2 (182 atoms in QM region), and LM (250 atoms in QM region). After optimization, the dihedral angles and glycosidic bond parameters of all three models are found to agree well with the crystal structure of α‐Gal bound M86 (Table S2) indicating that the size of the QM region does not have a significant effect on the description of the binding. Therefore, further analysis is based on the SM1 model, which is the one shown in Figure 5B (for the larger QM cores see Figure S9). Importantly, in the QM/MM models as well as in the crystal structures the Φ and Ψ angles of α‐Gal are in the region corresponding to the global minima we identified within the CREST/r^2^SCAN0‐D4 conformer ensembles for isolated α‐Gal.

From the X‐ray crystallography models, Langley *et al*. predicted that α‐Gal binds to its pocket mainly via two possible CH‐π interactions, eight hydrogen bonds with protein backbone and sidechains, and eight hydrogen bonds with six surrounding water molecules.^[15]^ In our QM/MM model, we also find that Tyr38 and Trp252 can participate in CH‐π interactions with the hydrogens from the A and B rings of α‐Gal, respectively. Depending only on the distances, we find that the backbone −NH group of Trp252 and the backbone carbonyls of Ser320 and His321 participate in three H‐bonds with the B‐ring, whereas the backbone carbonyls of His321 and Tyr322, the sidechain −OH and backbone carbonyl of Ser97 are responsible for five such bonds with the A‐ring (Figure 5). One intramolecular H‐bond (O_6_H⋅⋅⋅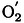 ) is present, the same discussed above in the context of conformational analysis. Although Langley *et al*. predicted eight possible H‐bonds between the disaccharide and bulk water, we found only six such bonding possibilities in our model with six out of seven bulk waters (w1–w6) from the first hydration sphere (Figure 5C). Among these, only one H‐bond is with the A‐ring, while the remaining five are with the B‐ring, which demonstrates that the two rings have substantially different exposure to the bulk. The seventh water molecule (w7) makes a hydrogen bond only with the backbone carbonyl of His321, which may play a role in positioning that residue to make two H‐bonds with the two rings.

In the following, we seek to move beyond structural descriptions and to better identify and quantify the interactions between α‐Gal, its pocket, and surrounding water molecules, using three different but complementary techniques: noncovalent interaction (NCI) analysis,^[81]^ local energy decomposition (LED)^[69]^ using the domain‐based local pair natural orbital coupled‐cluster method with single, double, and perturbative triple excitations DLPNO‐CCSD(T),[^64^, ^65^, ^66^, ^67^, ^68^] and the quantum theory of atoms in molecules (QTAIM)^[82]^ and related hydrogen bonding analysis methods.

**Figure 6:**
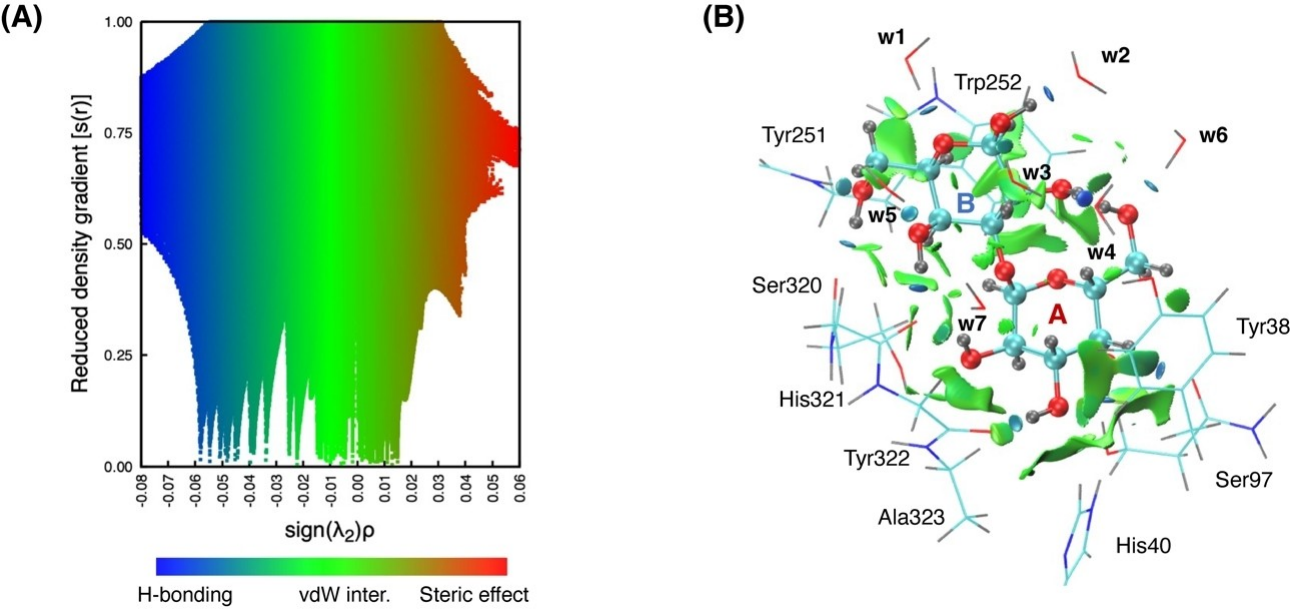
Non‐covalent Interaction (NCI) analysis of intermolecular interactions between α‐Gal and its surrounding using promolecular density. A) Reduced density gradient, s(**r**), is plotted against sign(λ_2_)ρ(**r**). Multiple low‐reduced gradient troughs in the λ_2_ <0 and λ_2_ ≈0 regions indicate the presence of multiple strong intermolecular H‐bond and stabilizing vdW interactions. Color code: blue for strong attractive interactions, green for weak van der Waals interactions, and red for strong steric effect. B) The s(**r**)=0.3 isosurface colored by sign(λ_2_)ρ(**r**) shows the positions of those intermolecular interactions in real space. α‐Gal is represented as ball‐and‐stick, whereas the surrounding residues from M86 and water molecules are shown as lines.

First, using NCIPLOT 4,^[71]^ we conducted NCI analysis to visualize intermolecular noncovalent interactions between α‐Gal and its environment, based on the analysis of the electron densities and their reduced gradients, s(**r**).^[81]^ Using a promolecular density, the dependence of reduced density gradient, and the sign of the second density Hessian eigenvalue (λ_2_) times the density, sign(λ_2_)ρ(**r**), is shown in Figure 6. We note that the s(**r**) vs. sign(λ_2_)ρ(**r**) intermolecular interactions plot is practically invariant to the size of the QM region in our QM/MM models (Figure S6). Several low‐density, low‐s(**r**) troughs appear in the λ_2_ <0 and λ_2_ ≈0 regions, indicative of attractive hydrogen‐bond interactions and weak vdW interactions. Careful inspection of the isosurface reveals that six waters (w1–w6) and the protein matrix of M86 make six and eight hydrogen bonds of different strengths, respectively (Figure 6 and Figure S5). The presence of broad surfaces between α‐Gal and the π‐rings of Trp252 and Tyr38 confirms that stabilizing vdW interactions are contributing to the binding, but additional dispersive interactions are present between α‐Gal and other residues in the binding pocket of M86.

In order to more precisely quantify the interactions between different fragments of our model, DLPNO‐CCSD(T)[^64^, ^65^, ^66^, ^67^, ^68^] based local energy decomposition^[69]^ analysis is performed within the present QM/MM framework. In this scheme, the DLPNO‐CCSD(T) level interaction energy is decomposed into a repulsive intramolecular energy term plus a number of physically meaningful energy terms such as electrostatic, exchange, and London dispersion interactions (Figure S7). Different fragment schemes can be utilized to determine the strength of the intermolecular interaction between any two fragments.

**Figure 7:**
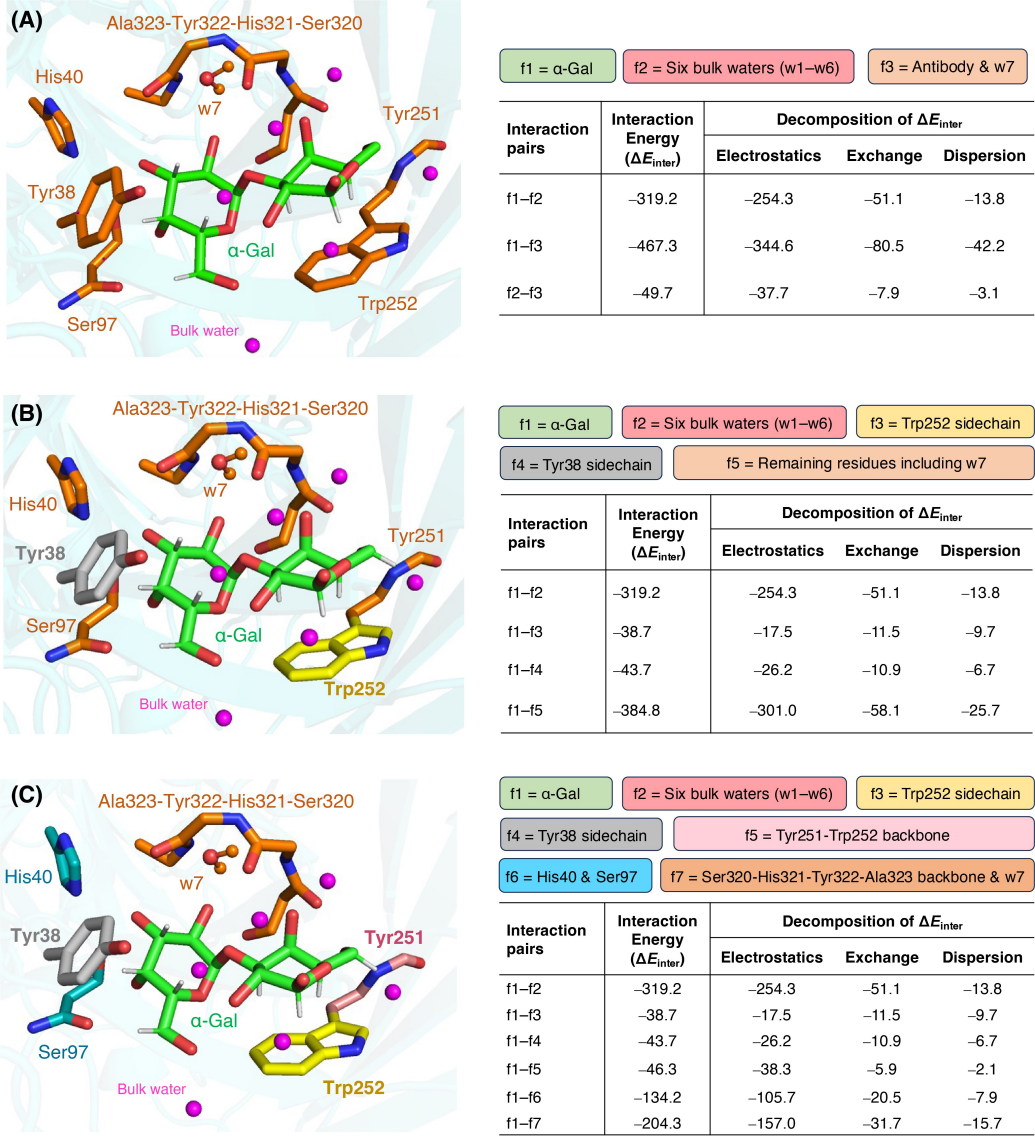
QM/MM local energy decompositions of the SM1 model of α‐Gal‐antibody complex using three fragmentation schemes (A, B, and C). At the left side of each panel, the α‐Gal and six bulk waters are shown in green and magenta colors, respectively. Total interaction energy (−Δ*E*_inter_ in kcal/mol) and its decomposition into electrostatic, exchange, and dispersion components are shown at the right side of in each panel.

As the first fragmentation scheme, we use α‐Gal, bulk water (w1–w6), and backbones and sidechains of the antibody and w7 as three different fragments (Figure 7A). The interaction energy between the first and last fragment is significantly larger than that between the first and second. The majority of that −Δ*E*_inter_ originates from the electrostatic component. Interestingly, only 9 % of total −Δ*E*_inter_ between α‐Gal and antibody fragments come from dispersion, whereas the contribution of that component in the −Δ*E*_inter_ from α‐Gal and bulk water is only ~4 %. This could be due to the fact that the majority of the α‐Gal surface is buried inside the M86 binding pocket. Analyzing the dispersion and CT components of the correlation contribution of the strong pairs to the interaction we find that the dominant part is the charge transfer from antibody and bulk water to α‐Gal (Figure S8).

In the next scheme, α‐Gal and bulk water are considered as two separate fragments, while the other three are the indole sidechain of Trp252, the phenol side chain of Tyr38, and the remaining atoms (Figure 7B). The third and fourth fragments are part of CH‐π interactions, but the last one interacts with the disaccharide mainly through hydrogen bonds. The interaction energy of α‐Gal with the Tyr38 side chain is slightly larger than its interaction with the Trp252 sidechain. However, the dispersion contribution of the former pair is smaller than the latter one. This may be due to the larger π‐surface of indole compared to phenol. The interaction energy originating from the protein‐disaccharide hydrogen bonds is significantly larger than two CH‐π interactions combined. For both residues involved in CH‐π interactions, α‐Gal→π‐ring charge transfer is almost equal to the π‐ring→α‐Gal charge transfer. For the remaining fragments, charge transfer towards α‐Gal is always dominant (Table S3).

Finally, in order to focus on the intermolecular H‐bonds in the M86‐α‐Gal complex model, the first four fragments of the second LED scheme are kept intact, but the last fragment is further divided into three: the Tyr251‐Trp252 backbone, His40 sidechain together with Ser97, and the remaining atoms (Figure 7C). The last fragment of this scheme is composed of the Ser320‐His321‐Tyr322‐Ala323 backbone and a water hydrogen bonded to His321. According to the NCI analysis, these three new fragments make one, three, and four hydrogen bonds, respectively, with the disaccharide (Figure S5). This is nicely reflected in the total interaction energies between α‐Gal and these new fragments (Figure 7C). As expected, the charge transfer contribution from a fragment to α‐Gal gradually increases with the increase of H‐bonds (Table S4).

In short, LED analysis using three different fragmentation schemes indicates that H‐bonds are the most critical structural component for α‐Gal binding. Two CH‐π interactions also play a nontrivial role. NCI analysis concurs that these intermolecular H‐bonds exist in varying strengths, which aligns with the H‐bond distances estimated in Ref. [15]. For a comprehensive understanding of the binding of α‐Gal to M86 quantitative analysis of each of these bonds will be conducted in the following.

As a final step, Bader's quantum theory of atoms in molecules (QTAIM),^[82]^ the core‐valence bifurcation (CVB) index based on the electron localization function (ELF),[^83^, ^84^] and H‐bond distances of the optimized structure were combined to fully characterize each HB interaction in our model. Within QTAIM, the electron density (ρ(**r**)) and real space functions such as energy density (H(**r**)), potential energy density (V(**r**)), Lagrangian kinetic energy density (G(**r**)), and the Laplacian of electron density (▿^2^ρ(**r**)) at the bond critical point (BCP) are used for quantitative analysis of H‐bonds.[^85^, ^86^, ^87^, ^88^, ^89^, ^90^, ^91^, ^92^] A more negative H_BCP_ corresponds to a stronger H‐bonding interaction. The |V_BCP_|/G_BCP_ ratio is an indicator of covalent character, while |V_BCP_|/G_BCP_ <1, 1 < |V_BCP_|/G_BCP_ >2, and 2 < |V_BCP_|/G_BCP_ correspond to closed‐shell, intermediate, and covalent interaction, respectively.^[86]^ Bond Degree (BD) is commonly used to assess bond strength or character and can be derived from the ratio H_BCP_/ρ_BCP_. For H_BCP_(**r**) >0, a more positive BD represents a weaker bond, whereas, for H_BCP_ <0, a more negative BD indicates a stronger bond. A positive CVB value is indicative of a weak H‐bond, which decreases with the increase of bond strength.[^83^, ^90^, ^93^] We used B3LYP‐D3(BJ) densities for QTAIM analysis and CVB index calculations. Emamian *et al*.^[85]^ showed that ρ_BCP_ values at the (3, −1) critical point have a nice liner relationship with the canonical CCSD(T) level hydrogen bond binding energies (BE), and the BEs for neutral H‐bonds can be accurately estimated using the equation BE≈−223.08×$\rho_{\mathrm{BCP}}\left(r\right)+0.7423$ . The topological and energetic properties of electron density at (3, −1) critical point for each of these bonds, together with the bond distance and CVB index are listed in Table S5.

As mentioned above, the protein matrix can form five and three hydrogen bonds with the A and B rings of α‐Gal, respectively (Figure 5). From the |V_BCP_|/G_BCP_ ratio it is evident that closed‐shell and intermediate interactions are present among these bonds. Consistent with the NCI analysis, the bond distance and CVB index also suggest that the two H‐bonds sharing the backbone carbonyl of His321 are of different strengths (3^rd^ and 4^th^ entries in Table S5). The B‐ring binds more than two times stronger with that carbonyl from His321 than the A‐ring. As the sidechain hydroxy group of Ser97 is already participating in a strong interaction with the α‐Gal O_4_, its interaction with O_5_ is weaker (Table S5). Among the eight, these two H‐bonds are the strongest and weakest bonds which bind α‐Gal with M86. From CVB indices and bond degrees, it is evident that all six hydrogen bonds between α‐Gal and bulk water are strong H‐bonds (Table S5). The strongest and weakest bonds are both with the B‐ring. The bond distance and CVB index of the only intramolecular H‐bond in α‐Gal (i. e., O_6_H⋅⋅⋅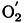 ) indicate that it belongs to the weak interaction regime. From the CREST/r^2^SCAN0‐D4 ensemble, among the structures which have only one intramolecular H‐bond, the O_6_H⋅⋅⋅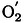 bond is present in only three instances.

In short, from QTAIM analysis, CVB indices, and bond distances we find that although the A‐ring is involved in two extra hydrogen bonds than the B‐ring, these two are relatively weak bonds. All H‐bonds with the waters from first hydration sphere belong instead to the strong interaction regime. These results complement the conclusions of the NCI and LED analyses and complete the quantitative description of the specific interactions involved in α‐Gal binding to the antibody.

## Conclusions

In this study we investigated the conformational landscape of the biologically relevant disaccharide galactose‐α‐1,3‐galactose (α‐Gal) and the interaction of the molecule with the M86 antibody as the initiating structural event of the anaphylactic response that underlies the α‐Gal syndrome. Form an extensive survey of the conformational profile of α‐Gal in both explicit and implicit solvation, we conclude that the structures from classical MD trajectory have one major population island, whereas the CREST/DFT protocol identifies two prominent population clusters in the Φ vs. Ψ plot. Only positive Φ values along a relatively narrow range are observed, consistent with this conformational element being particularly rigid. The largest number of conformers exists in the negative Ψ region. Although the Ψ angles among the xTB‐sampled rotamers are more dispersed, after optimizing them with r^2^SCAN0‐D4 we again obtain localization into two distinct clusters. The lowest energy conformer from our CREST/r^2^SCAN0‐D4 protocol always stays in the “negative Ψ” region. Among these structures, the most stable one (Φ=102.8°, Ψ=−160.0°) is 1.33 kcal/mol lower in energy than the local minimum from the “positive Ψ” island (Φ=85.7°, Ψ=61.1°). The number of intramolecular hydrogen bonds is affected by the treatment of solvation, which confirms the need to treat the conformational problem using multiple complementary methodological approaches. It should be borne in mind that the MD and CREST should not be considered equivalent in terms of conformational exhaustivity. Given that they represent fundamentally distinct approaches to the problem of sampling, they should rather be viewed as distinct but complementary tools, each providing different insights into the behavior and conformational complexity of a given system. Our use of modern DFT functionals to obtain final energetic ordering was shown to be computationally efficient and also a necessary step to refine the energetics of the initial CREST results. Higher‐level wave function methods can also be employed to this end,[^94^, ^95^, ^96^, ^97^] even for large scale applications as already demonstrated with local coupled cluster approaches.^[98]^

QM/MM investigation of a selected antibody–α‐Gal complex established that the Φ and Ψ dihedrals and glycosidic bond parameters agree well the reported crystal structure independently of the size of the QM region. The binding pose is not identical but correlates well with the most populated conformers/rotamers identified in the first stage of the study. The B ring of α‐Gal is substantially more exposed to the bulk compared to the A ring, which instead is more extensively involved in interactions with the M86 antibody. Intermolecular NCI analysis indicates the existence of a variety of hydrogen‐bonds and weak vdW interactions. DLPNO‐CCSD(T) level energy decomposition analysis suggests that H‐bond interactions are significantly more dominant than the CH‐π interactions with Tyr38 and Trp252 for the binding of α‐Gal to the antibody. From QTAIM and other quantitative analysis of the H‐bonds between α‐Gal and the protein or bulk waters, we establish their relative strength and show that they can vary from a strong to a very weak interaction. Although the energies of all H‐bonds between α‐Gal and surrounding water differ slightly, they all belong to a strong interaction regime. Overall, the present study elucidates the conformational profile of α‐Gal from multiple computational perspectives and provides an atomic‐level quantification of the distinct directional and non‐covalent interactions involved in antibody binding.

## Conflict of Interests

The authors declare no conflict of interest.

1

## Supporting information

As a service to our authors and readers, this journal provides supporting information supplied by the authors. Such materials are peer reviewed and may be re‐organized for online delivery, but are not copy‐edited or typeset. Technical support issues arising from supporting information (other than missing files) should be addressed to the authors.

Supporting Information

## Data Availability

The data that support the findings of this study are available in the supplementary material of this article.
