# Supplementary material for: Conformational Profile of Galactose‐α‐1,3‐Galactose (α‐Gal) and Structural Basis of Its Immunological Response
Source: Chemistry. 2025 Mar 18;31(22):e202500050. doi: 10.1002/chem.202500050 (PMC12015390; doi:10.1002/chem.202500050)
Supplement: Supplementary file 1 — Supporting Information [file CHEM-31-e202500050-s001.pdf]

Chemistry–A European Journal

Supporting Information

Conformational Profile of Galactose-α-1,3-Galactose (α-Gal) and Structural Basis of Its Immunological Response

Golokesh Santra and Dimitrios A. Pantazis*

Supplementary Information

for

Conformational Profile of Galactose-α-1,3-Galactose (α-Gal) and Structural Basis of Its Immunological Response

Golokesh Santra and Dimitrios A. Pantazis*

Max-Planck-Institut für Kohlenforschung, Kaiser-Wilhelm-Platz 1, 45470 Mülheim an der Ruhr, Germany

* Email: dimitrios.pantazis@kofo.mpg.de

I. Computational Methodology

Molecular dynamics simulations

Molecular dynamics (MD) simulations were performed using the AMBER program together with the generalizable biomolecular force field for carbohydrates, GLYCAM06,[1-2] and the non-polarizable, 4-point, 3-charge rigid water model, OPC.[3-4] Hydrogens were constrained with the SHAKE algorithm.[5-6] The simulations were carried out in the isothermal-isobaric ensemble (NPT) at a temperature of 300 K and a pressure of 1 atm. The Langevin thermostat and Monte Carlo barostat were used for temperature and pressure control, respectively.[7-8] The non-bonded cutoff was set to 8.0 Å. A truncated octahedron box with 1042 water molecules was used for solvating α-Gal. As pyranose ring puckering occurs at the microsecond timescale, sub-microsecond simulation may not be enough to fully explore the conformational space of α-Gal,[9-12] therefore after solvent minimization and a short equilibration (1 ns), a production MD simulation was carried out for 1 μs. From the total trajectory, 100 000 frames were extracted using CPPTRAJ for further analysis.[13] The single GPU version of PMEMD was employed throughout.

CREST and DFT calculations

The unique rotamers for α-Gal were generated using Grimme's Conformer–Rotamer Ensemble Sampling Tool (CREST 3.0).[14-15] The CREST calculations were performed by employing the semi-empirical tight-binding method, GFN2-xTB,[16] and the analytical linearized Poisson-Boltzmann (ALPB) implicit solvation model.[17] A 0–6 kcal/mol relative energy range was employed for the CREST conformer generation. Water was used as solvent. The semiempirical method GFN2-xTB was chosen over GFN1-xTB, because it has been shown to provide more accurate sugar conformers and rotamers.[16] It is known that most semiempirical quantum mechanical (SQM) methods tend to underestimate conformational energies, especially for high-energy conformers (see ref. [16] and references therein). When the CREST-generated α-Gal conformer energies were re-evaluated using the more accurate DFT functional r^2SCAN0-D4,[18] the above mentioned 6 kcal/mol energy gap increased to 14.4 kcal/mol. In the next step, geometry optimizations were performed using the implicit solvation model SMD (Solvation Model based on Density),[19] and three DFT functionals, R^2SCAN-3c,[20] R^2SCAN-D4[21] and R^2SCAN0-D4.[18] DefGrid3 integration grid, TightOpt convergence threshold, and the RIJCOSX approximation were also employed.[22] Except for the low-cost R^2SCAN-3c method, def2-TZVPP basis sets with corresponding def2/J auxiliary basis sets were used for the other two functionals.[23] This entire

process, involving CREST rotamer generation followed by a DFT optimization, is referred to as CREST/DFT throughout this paper. All electronic structure calculations were performed using ORCA[24] and analysis of the results relied on a collection of python codes developed in-house. The root-mean-square deviations (RMSDs) were calculated using Kromann's rmsd-1.5.1 code, which uses the Kabsch algorithm for rotation between two Cartesian coordinates to obtain the minimal RMSD.[25-26]

The α-Gal–antibody complex

After extracting the coordinates from the Protein Data Bank, the missing residues of 7uen (except the terminal ones) were modelled using MODELLER.[27] The resultant structure along with the crystallographic waters were solvated in a 15 Å cubic box under periodic boundary condition. The simulation was performed using the FF19SB protein force field,[28] the TIP3P water model,[29] and GAFF2 for α-Gal using restrained electrostatic potential (REsP) charges refitted using Multiwfn.[30] After solvent minimization, a very short (0.7 ns) NPT simulation was carried out by employing a restraint of 5 kcal/mol/Å^2 on the protein and ligand complex. The Langevin thermostat and Monte Carlo barostat were used for temperature and pressure control, respectively.[7-8] Atomic coordinates were saved every 10 ps. After visual inspection of the last few frames, the second-last was selected as starting structure for the QM/MM model preparation.

Three models with different QM and active regions are considered: two relatively smaller models (SM1 and SM2), and a larger model (LM).

1. **SM1**. The QM region of this model is composed of α-Gal, side chains of Tyr38 and His40, complete Ser97 residue except its backbone NH together with the backbone NH of Thr98, only the backbone carbonyl of Tyr251 with the side chain and backbone NH of Trp252, complete Ser320 residue except its backbone NH, only the His321-Tyr322 backbone, complete Ala323 except its backbone carbonyl, six water molecules (w1-w6) from the first hydration sphere of α-Gal, and one extra water (w7) which makes a hydrogen bond with the backbone carbonyl of His321. Without the link atoms, the QM and active regions have 156 and 707 atoms, respectively.
2. **SM2**. The QM region of this model contains all the QM atoms of SM1. The only difference is instead of only the His321-Tyr322 backbone, we consider complete His321 and Tyr322 residues. Possible π–π interaction (crystallographic distance between two rings is 4.1 Å) of the side chains of His321 and Tyr322 may play a role in the orientation of the backbone

carbonyl of His321, which makes two H-bonds with the ligand. Without the link atoms, the QM region has 182 atoms. The size of the active region is the same as SM1.

3. **LM**. The QM region of this model contains all the QM atoms of SM2, the side chain of Tyr275, and 12 extra waters molecules present in the second hydration sphere of α-Gal. Instead of the backbone carbonyl of Tyr251, we consider the complete residue without its backbone NH. Without the link atoms, the QM and active region has 250 and 1095 atoms, respectively.

All three models include the residues identified as critical for the CH–π interactions and hydrogen bonds between α-Gal and its binding pocket in the M86 antibody.[31]

The QM/MM optimizations of the above models were performed using the BP86 functional,[32] def2-TZVP basis set,[23] and def2/J auxiliary basis set. For the MM region, we have used the same forcefield parameters as the MD calculation. Single-point QM/MM calculations were carried out using the B3LYP functional,[33-34] def2-TZVP basis set, and DefGrid3 integration grid. The DLPNO-CCSD(T)[35-39] (domain based local pair natural orbital coupled-cluster method with single, double, and perturbative triple excitations) local energy decomposition (LED) analysis[40] was conducted by employing the def2-TZVP basis set,[23] TightPNO thresholds, and the Pipek–Mezey scheme for localizing pair natural orbitals (PNOs).[41] All QM/MM calculations are performed using ORCA[24] and further analysis was conducted using Multiwfn[30] and NCIPLOT4.[42] Figures are prepared using PyMOL (Version 2.5.5, Schrödinger, LLC), VMD,[43] gnuplot 6.0, and our own python codes.

II. Figures and Tables:

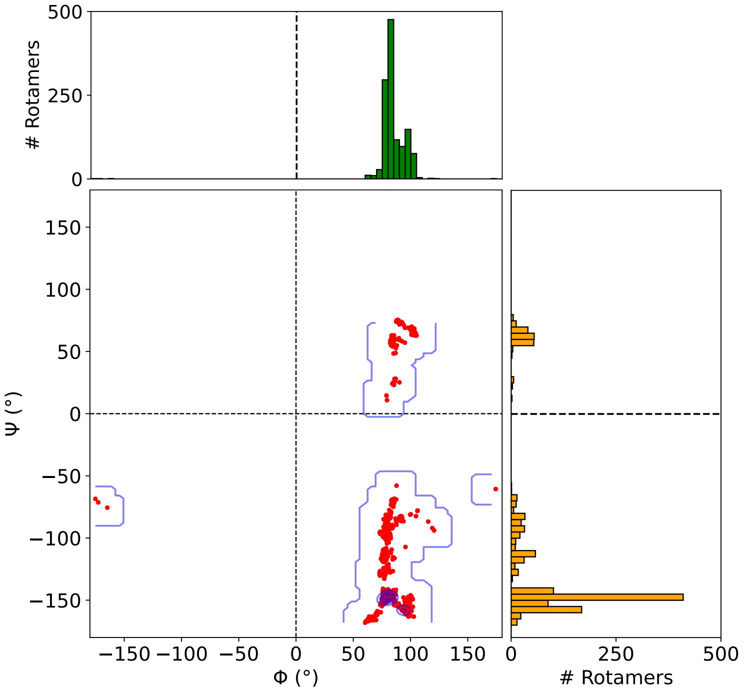

Figure S1: The Φ vs. Ψ scatter plot of the r²SCAN-3c optimized 1270 rotamers.

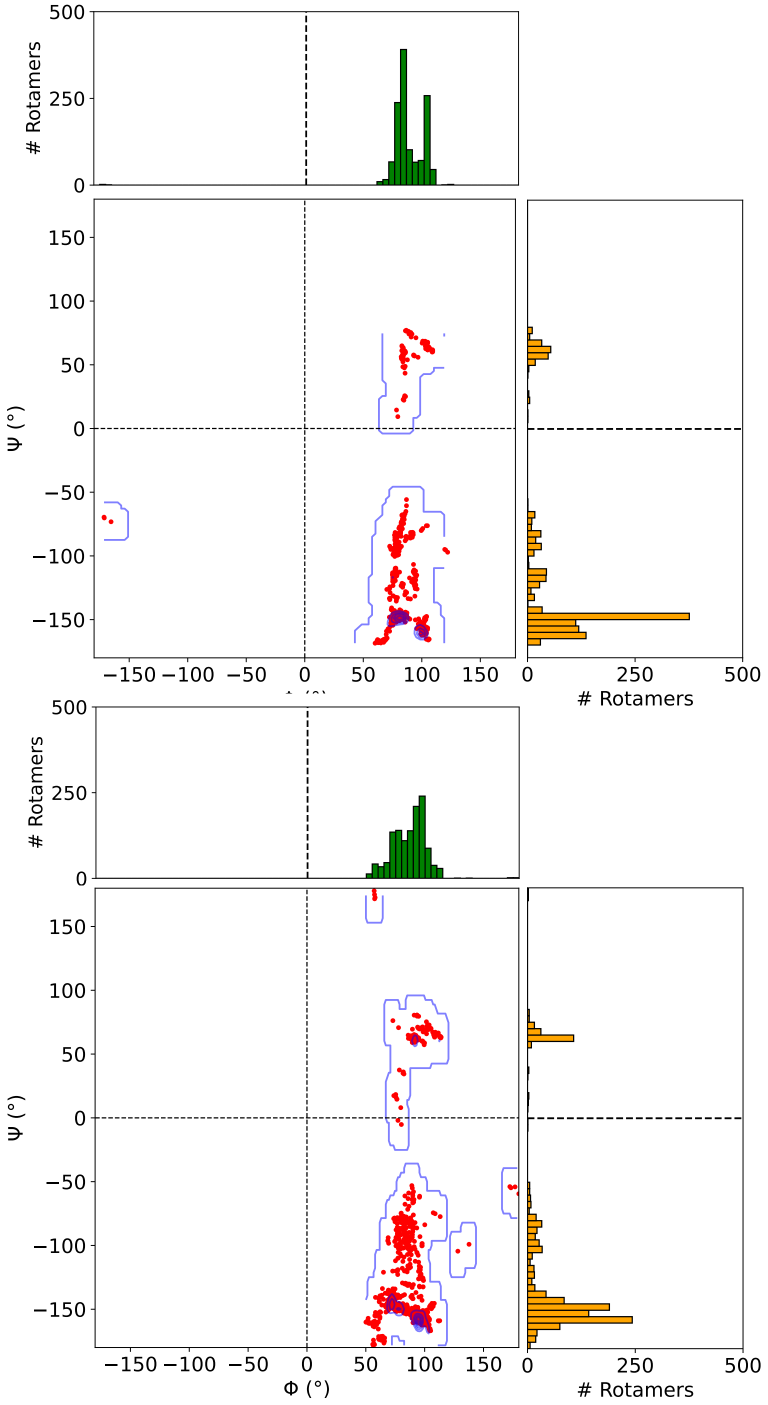

Figure S2: The Φ vs. Ψ scatter plot of the r^{2}SCAN-D4 optimized 1270 rotamers, using implicit SMD (water) solvation model (top) and no solvent (bottom).

(a)

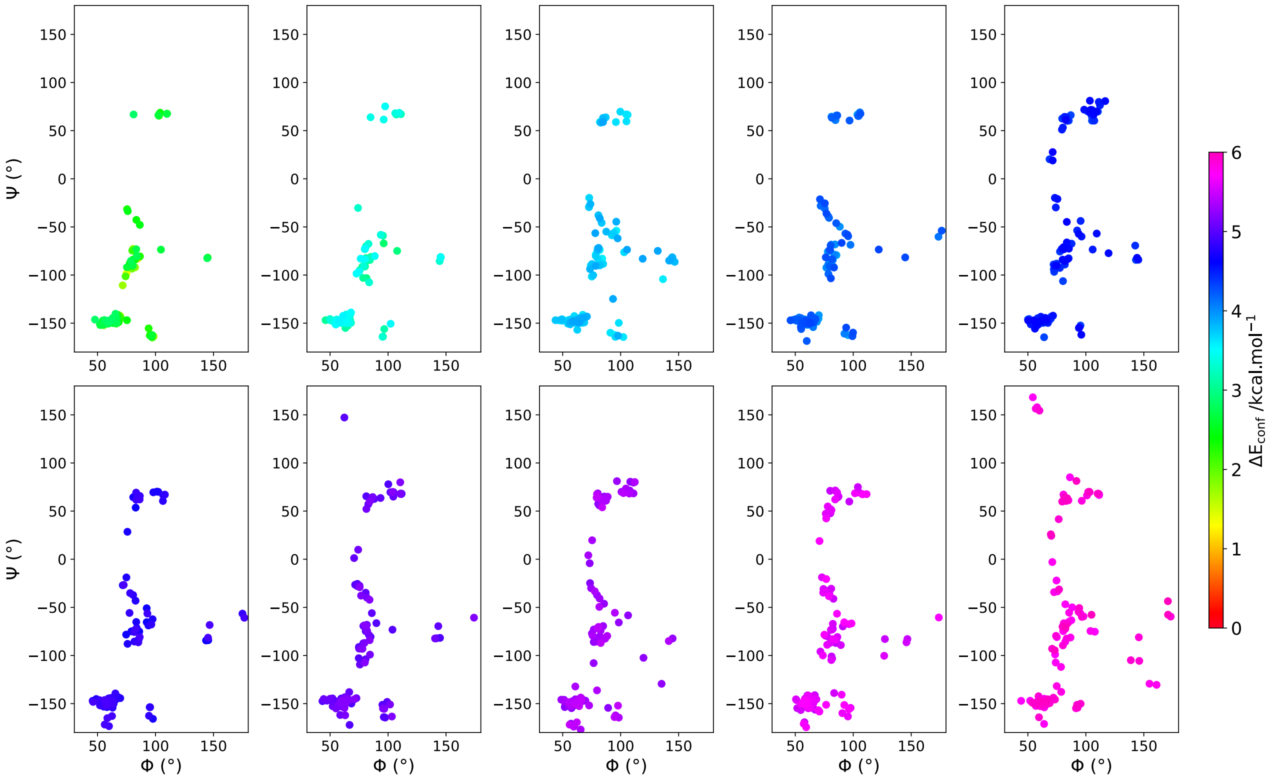

(b)

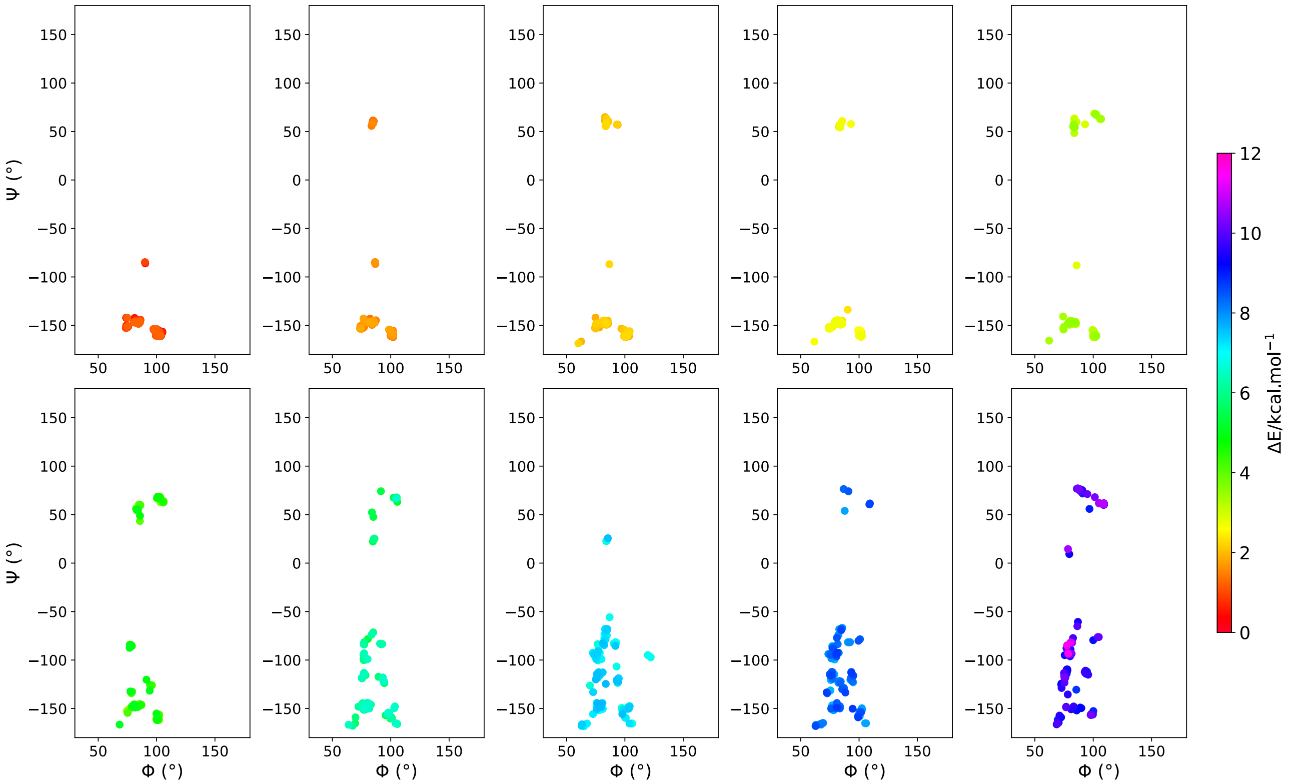

Figure S3: The CREST (a) and r^{2}SCAN-D4 (b) relative energies for the 1270 rotamers with respect to the most stable structure. Each panel contains 127 rotamers.

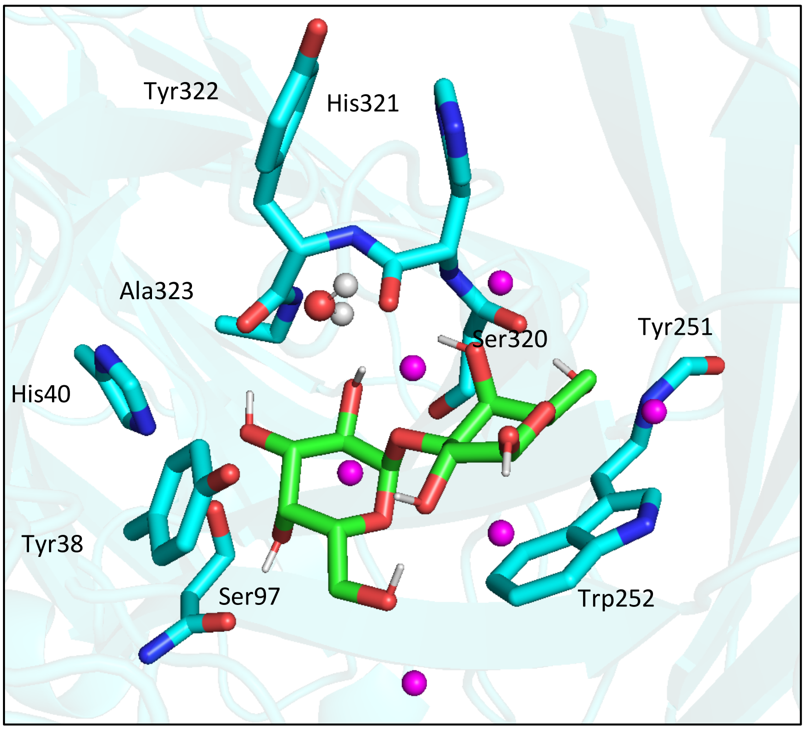

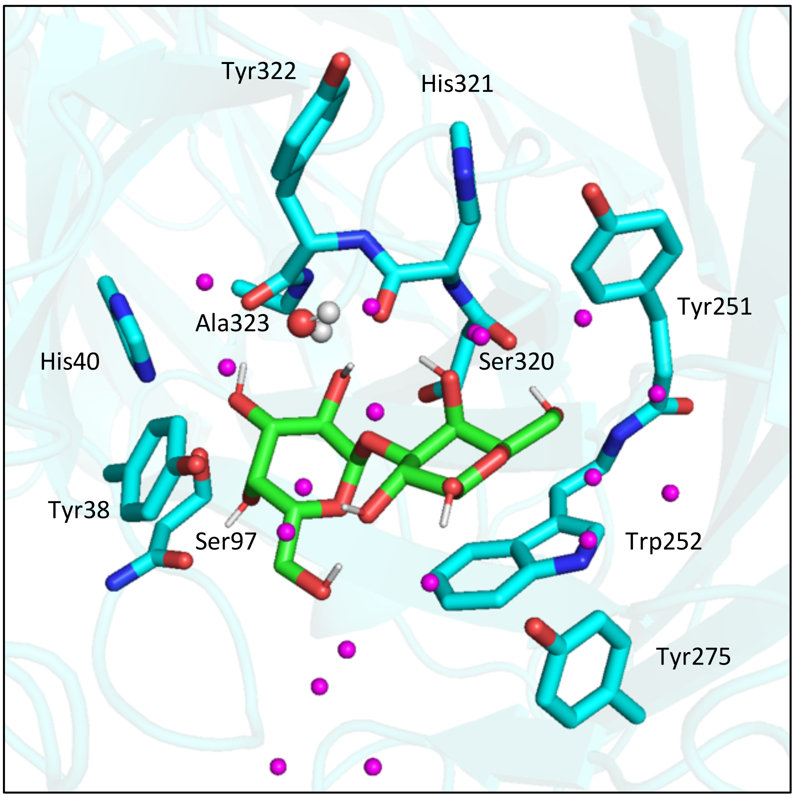

Figure S4: The optimized QM core of the SM2 (top) and LM (bottom) models. The surrounding residues from the M86 antibody are shown in cyan, the α-Gal is shown in green, and the bulk water oxygens are shown as magenta spheres. The water molecule which binds to the backbone carbonyl of His321 is shown in ball-stick representation.

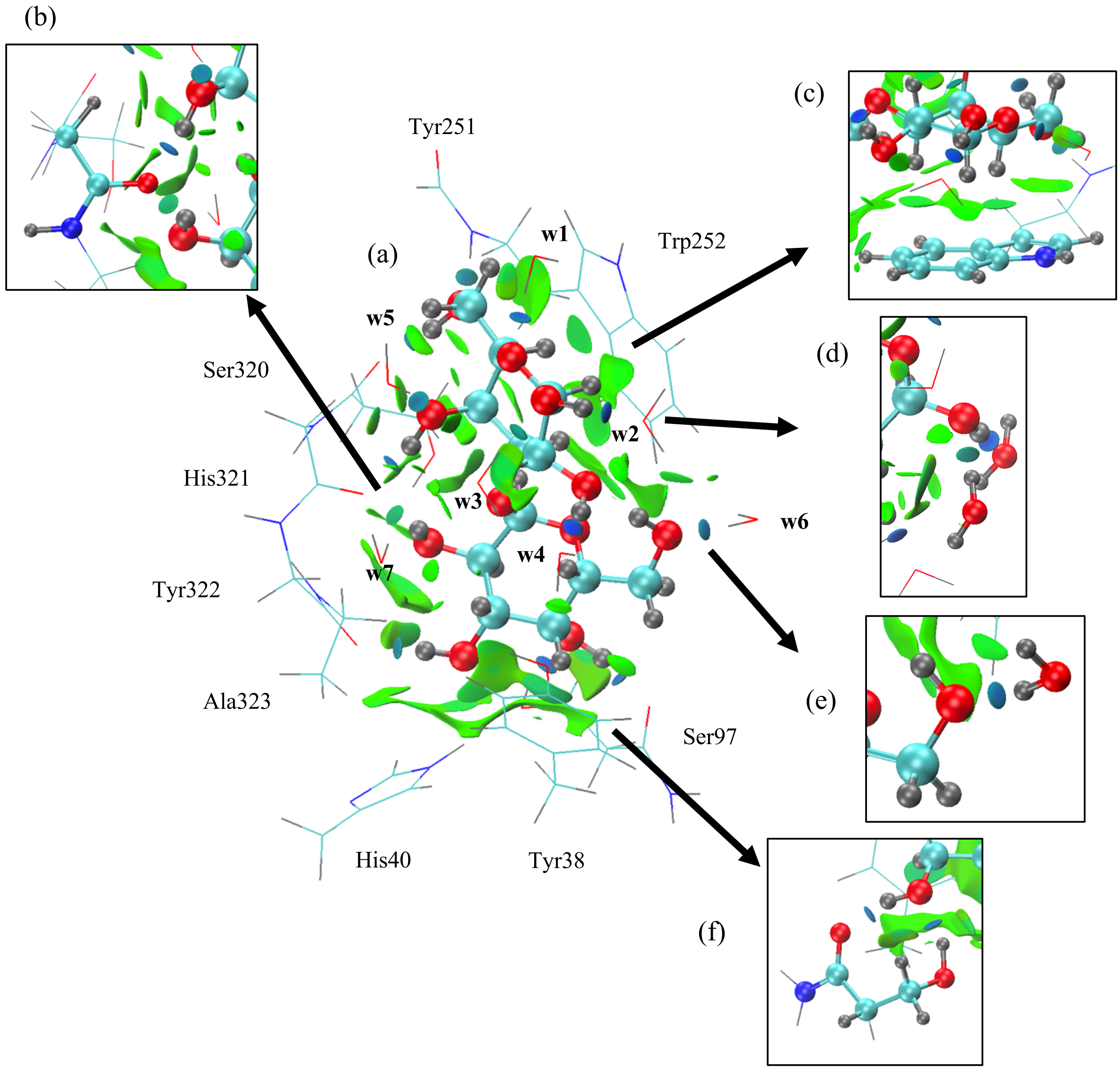

Figure S5: Noncovalent interaction (NCI) analysis of intermolecular interactions between α-Gal and its surroundings using a promolecular density. (a) The s(**r**)=0.3 isosurface colored by sign(λ_2)ρ(**r**). α-Gal is represented as ball-and-stick, whereas the surrounding residues from M86 and water molecules are represented as lines. The M86 residues and waters are labeled in normal and bold fonts, respectively; (b) two HBs sharing the backbone carbonyl of His321; (c) CH–π interactions between α-Gal and Trp252 side chain; (d-e) H-bonds of different strength between waters and α-Gal; and (f) H-bonds between the backbone carbonyl and side chain –OH of Ser97. Color code: blue for strong attractive interactions ($\lambda_2 < 0$) and green for weak van der Waals interactions ($\lambda_2 \approx 0$).

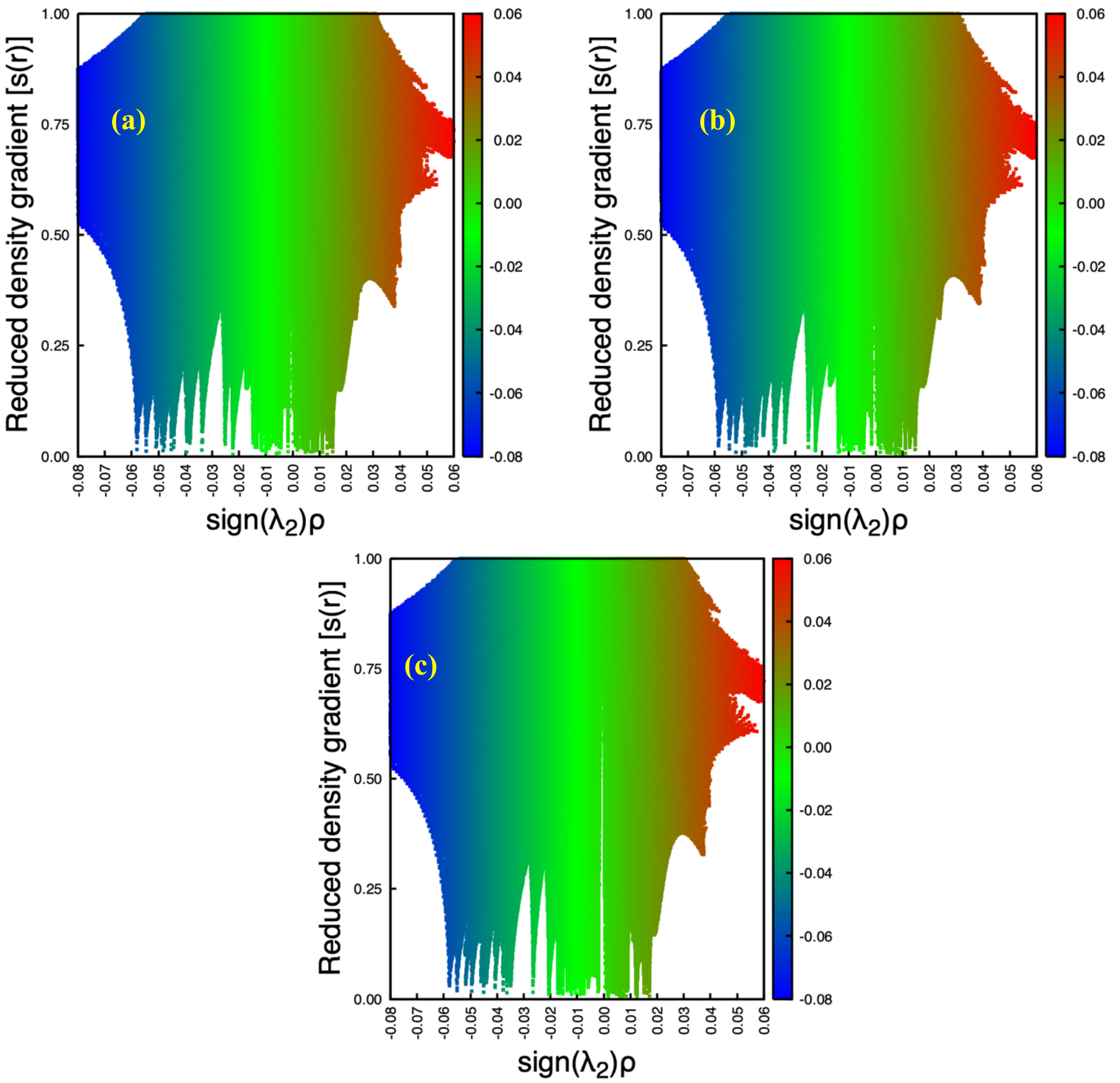

Figure S6: The s(**r**) vs. sign(λ_2)ρ(**r**) plot from noncovalent interaction (NCI) index analysis of three QM/MM models: a) SM1, b) SM2, and c) and LM. Color code: blue for strong attractive interactions ($\lambda_2 < 0$), green for weak van der Waals interactions ($\lambda_2 \approx 0$) and red for strong repulsive interactions ($\lambda_2 > 0$).

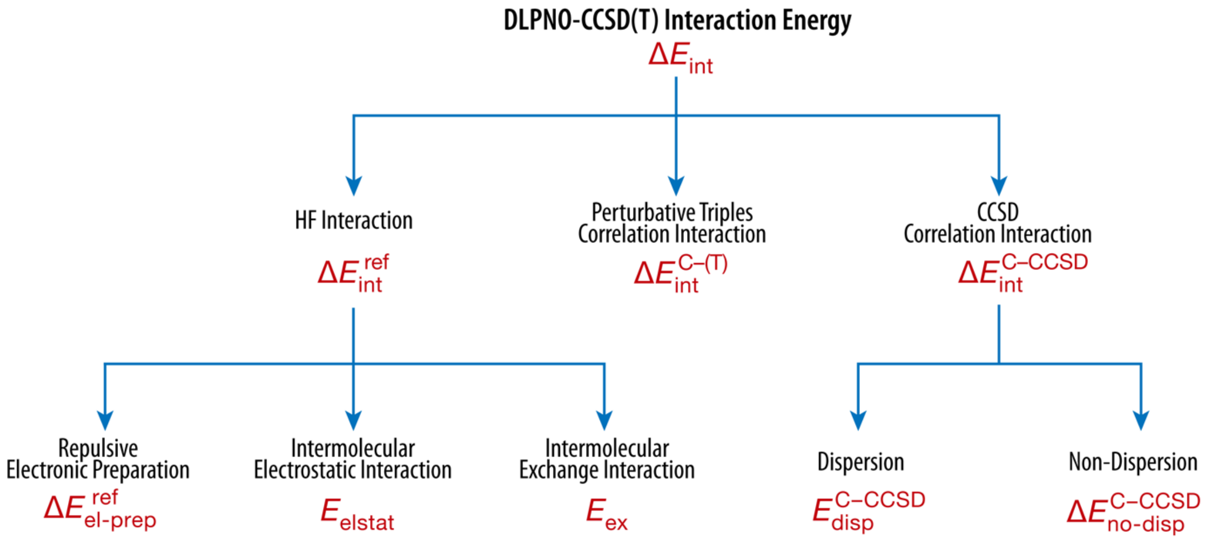

Figure S7: Schematic representation of DLPNO-CCSD(T) based local-energy decomposition (LED).

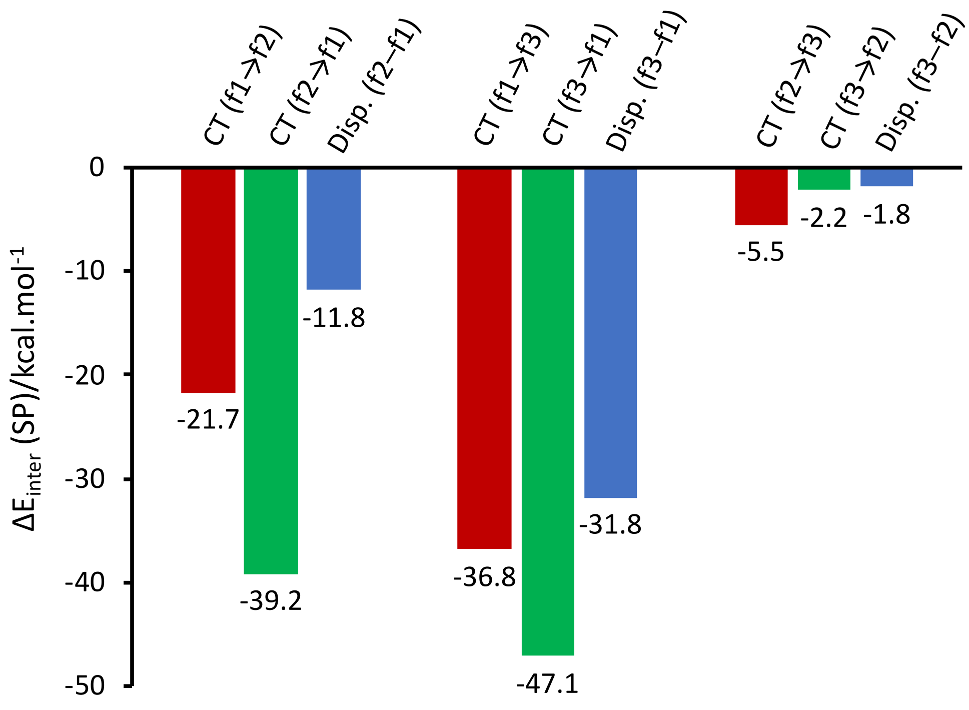

Figure S8: Decomposition of CCSD strong pairs for the three fragments in the first LED scheme: in this fragmentation approach, we use α-Gal, bulk water (w1–w6), and backbones and sidechains of the antibody and w7 as three different fragments. For each interaction pair, charge transfer (CT) in both directions and dispersion components are analyzed.

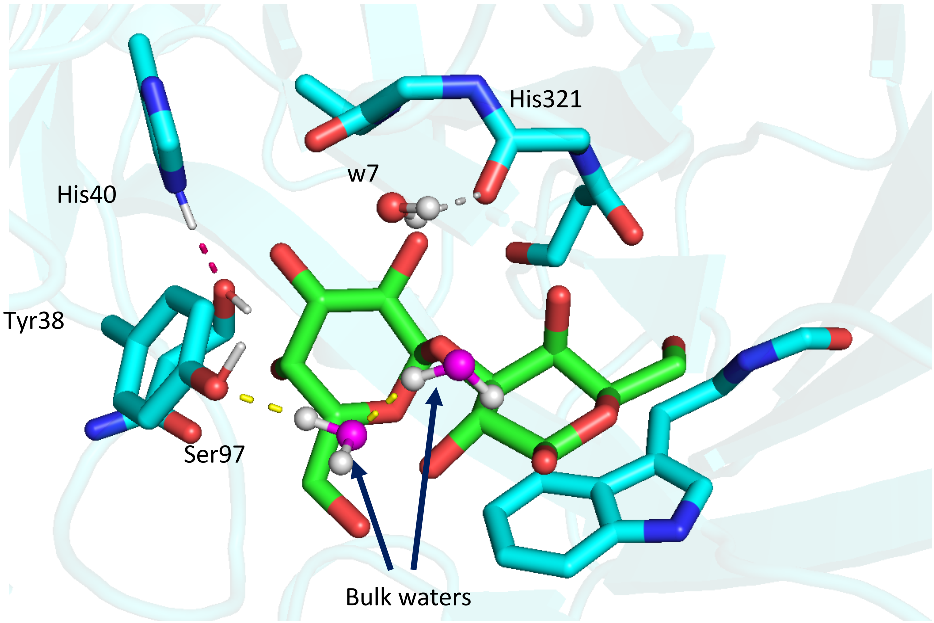

Figure S9: Four extra intermolecular hydrogen bonds found in the optimized QM core of the SM1 model. The hydrogen bond between bulk water and protein matrix (Tyr38) is shown is yellow. Intramolecular H-bonds between the His40 and Ser97 residue of the antibody shown in red. The H-bond between w7 and backbone carbonyl of His321 is shown in grey.

Table S1: Percentage of structures in five ensembles with specific number of hydrogen bonds. Among the five methods first only MD involves explicit solvation, whereas the remaining four uses implicit solvation models in calculations.

**# Hydrogen Bonds**	**MD (1μs)**	**CREST/ GFN2-xTB**	**CREST/ r^2SCAN-D4**	**CREST/ r^2SCAN0-D4**	**CREST/ r^2SCAN-D4 (no solvent)**
0	72.1	2.8	15.6	16.5	7.1
1	26.1	41.5	39.4	39.7	27.2
2	1.7	42.0	33.0	33.4	37.5
3	<0.1	13.0	11.8	10.2	25.0
4	–	0.6	0.2	0.2	3.1
5	–	0.1	–	–	0.2

Table S2: Comparison of Φ, Ψ, and glycosidic bond parameters among the crystallographic structure (PDB ID: 7uen) and three QM/MM models of a-Gal bound to the M86 antibody, with increasing size of the QM region.

	Φ (°)	Ψ (°)	*glycosidic bond parameters*		
			$\angle C_1-O_1-C_3'$ (°)	C_1-O_1 (Å)	O_1-C_3' (Å)
7uen[31]	77.6	–137.7	112.5	1.37	1.46
SM1	72.2	–139.3	111.9	1.42	1.44
SM2	72.5	–139.6	112.0	1.42	1.44
LM	71.7	–137.4	112.5	1.43	1.44

Table S3: Decomposition of CCSD strong pairs for the interaction between α-Gal (f1) and the remain four fragments in the second LED scheme. The contributions from each component [i.e., $\Delta\Delta E_{inter}$ (SP)] are given in kcal/mol.

**f*n***	**CT (f1→f*n*)**	**CT (f*n*→f1)**	**Dispersion (f*n*-f1)**
**f2**	-21.71	-39.19	-11.77
**f3**	-4.52	-4.98	-7.00
**f4**	-5.47	-4.79	-5.00
**f5**	-26.19	-37.55	-19.74

Table S4: Decomposition of CCSD strong pairs for the interaction between α-Gal (f1) and the remain six fragments in the third LED scheme. The contribution from each component [i.e., $\Delta\Delta E_{inter}$ (SP)] are given in kcal/mol.

f*n*	CT (f1→f*n*)	CT (f*n*→f1)	Dispersion (f*n*-f1)
**f2**	-21.69	-39.18	-11.76
**f3**	-4.51	-4.95	-7.01
**f4**	-5.53	-4.79	-4.99
**f5**	-5.96	-0.58	-1.75
**f6**	-12.60	-9.51	-6.25
**f7**	-7.66	-27.44	-11.70

Table S5: Topological and energetic properties of electron density at the bond critical point (ρ(r)), core-valence bifurcation (CVB) index, and distance hydrogen bonds present in SM1 model. First eight hydrogen bonds are between α-Gal and protein matrix, next six are between α-Gal and bulk waters. The 15th hydrogen bond is the only intramolecular HB present in M86-bound α-Gal (green shade). The 16th H-bond is between w7 and the His321 backbone carbonyl. The last three bonds (16-19) are additional H-bonds in protein matrix and bulk solvent not involving α-Gal. The H-bonds involving A and B-ring are in grey and yellow shades, respectively.

#HBs[a]	ρ(r) (a.u.)	H(r) (a.u.)	$\frac{\|V(r)\|}{G(r)}$	BD[b]	BE[c] (kcal·mol^{-1})	CVB Index	d_{HB} (Å)
**1**	0.0314	0.0002	0.991	0.0076	–6.26	–0.0031	1.84
**2**	0.0358	–0.0015	1.043	–0.0407	–7.24	–0.0038	1.74
**3**	0.0440	–0.0067	1.180	–0.1514	–9.07	–0.0526	1.71
**4**	0.0185	0.0026	0.837	0.1385	–3.39	0.0385	2.05
**5**	0.0329	–0.0016	1.061	–0.0502	–6.59	–0.0149	1.82
**6**	0.0469	–0.0086	1.225	–0.1839	–9.73	–0.0694	1.71
**7**	0.0420	–0.0050	1.138	–0.1200	–8.63	–0.0382	1.72
**8**	0.0098	0.0023	0.728	0.2363	–1.44	0.0773	2.39
**9**	0.0429	–0.0065	1.183	–0.1515	–8.84	–0.0524	1.73
**10**	0.0515	–0.0116	1.280	–0.2256	–10.75	–0.0904	1.66
**11**	0.0284	–0.0001	1.006	–0.0051	–5.59	–0.0032	1.90
**12**	0.0548	–0.0125	1.274	–0.2281	–11.48	–0.0926	1.62
**13**	0.0331	–0.0015	1.054	–0.0455	–6.65	–0.0106	1.81
**14**	0.0387	–0.0042	1.128	–0.1072	–7.90	–0.0341	1.76
**15**	0.0249	0.0009	0.953	0.0380	–4.81	0.0109	1.93
**16**	0.0114	0.0023	0.733	0.2027	–1.79	0.0563	2.27
**17**	0.0288	–0.0002	1.008	–0.0070	–5.68	–0.0006	1.89
**18**	0.0204	0.0029	0.842	0.1402	–3.80	0.0431	2.01
**19**	0.0241	0.0013	0.936	0.0525	–4.63	0.0159	1.97

[a]For the visualization of #HBs (1-8 and 15) see Figure 5B in the main text; for #HBs (9-14) see Figure 5C in the main text; for #HBs (16-19) see Figure S9.

[b] Bond degree (BD) = H_{BCP}/ρ_{BCP}

[c] According to ref. [44], bond energy (BE) ≈ $-223.08 \times \rho_{BCP}(\mathbf{r}) + 0.7423$

III. References

[1] K. N. Kirschner, A. B. Yongye, S. M. Tschampel, J. González-Outeiriño, C. R. Daniels, B. L. Foley, R. J. Woods, *J. Comput. Chem.* **2008**, *29*, 622-655.

[2] A. Plazinska, W. Plazinski, *J. Chem. Theory Comput.* **2021**, *17*, 2575-2585.

[3] S. Izadi, R. Anandakrishnan, A. V. Onufriev, *J. Phys. Chem. Lett.* **2014**, *5*, 3863-3871.

[4] R. Salomon-Ferrer, D. A. Case, R. C. Walker, *WIREs Comput. Mol. Sci.* **2013**, *3*, 198-210.

[5] S. Miyamoto, P. A. Kollman, *J. Comput. Chem.* **1992**, *13*, 952-962.

[6] J.-P. Ryckaert, G. Ciccotti, H. J. C. Berendsen, *J. Comp. Phys.* **1977**, *23*, 327-341.

[7] R. J. Loncharich, B. R. Brooks, R. W. Pastor, *Biopolymers* **1992**, *32*, 523-535.

[8] J. Åqvist, P. Wennerström, M. Nervall, S. Bjelic, B. O. Brandsdal, *Chem. Phys. Lett.* **2004**, *384*, 288-294.

[9] B. M. Sattelle, J. Shakeri, A. Almond, *Biomacromolecules* **2013**, *14*, 1149-1159.

[10] B. M. Sattelle, S. U. Hansen, J. Gardiner, A. Almond, *J. Am. Chem. Soc.* **2010**, *132*, 13132-13134.

[11] O. Guvench, D. Martin, M. Greene, *Int. J. Mol. Sci.* **2022**, *23*, 473.

[12] B. M. Sattelle, B. Bose-Basu, M. Tessier, R. J. Woods, A. S. Serianni, A. Almond, *J. Phys. Chem. B* **2012**, *116*, 6380-6386.

[13] D. A. Case, H. M. Aktulga, K. Belfon, D. S. Cerutti, G. A. Cisneros, V. W. D. Cruzeiro, N. Forouzesh, T. J. Giese, A. W. Götz, H. Gohlke, S. Izadi, K. Kasavajhala, M. C. Kaymak, E. King, T. Kurtzman, T.-S. Lee, P. Li, J. Liu, T. Luchko, R. Luo, M. Manathunga, M. R. Machado, H. M. Nguyen, K. A. O'Hearn, A. V. Onufriev, F. Pan, S. Pantano, R. Qi, A. Rahnamoun, A. Risheh, S. Schott-Verdugo, A. Shajan, J. Swails, J. Wang, H. Wei, X. Wu, Y. Wu, S. Zhang, S. Zhao, Q. Zhu, T. E. Cheatham, III, D. R. Roe, A. Roitberg, C. Simmerling, D. M. York, M. C. Nagan, K. M. Merz, Jr., *J. Chem. Inf. Model.* **2023**, *63*, 6183-6191.

[14] P. Pracht, F. Bohle, S. Grimme, *Phys. Chem. Chem. Phys.* **2020**, *22*, 7169-7192.

[15] P. Pracht, S. Grimme, C. Bannwarth, F. Bohle, S. Ehlert, G. Feldmann, J. Gorges, M. Müller, T. Neudecker, C. Plett, S. Spicher, P. Steinbach, P. A. Wesołowski, F. Zeller, *J. Chem. Phys.* **2024**, *160*, 114110.

[16] C. Bannwarth, S. Ehlert, S. Grimme, *J. Chem. Theory Comput.* **2019**, *15*, 1652-1671.

[17] G. Sigalov, A. Fenley, A. Onufriev, *J. Chem. Phys.* **2006**, *124*, 124902.

[18] M. Bursch, H. Neugebauer, S. Ehlert, S. Grimme, *J. Chem. Phys.* **2022**, *156*, 134105.

[19] A. V. Marenich, C. J. Cramer, D. G. Truhlar, *J. Phys. Chem. B* **2009**, *113*, 6378-6396.

[20] S. Grimme, A. Hansen, S. Ehlert, J.-M. Mewes, *J. Chem. Phys.* **2021**, *154*, 064103.

[21] S. Ehlert, U. Huniar, J. Ning, J. W. Furness, J. Sun, A. D. Kaplan, J. P. Perdew, J. G. Brandenburg, *J. Chem. Phys.* **2021**, *154*, 061101.

[22] B. Helmich-Paris, B. de Souza, F. Neese, R. Izsák, *J. Chem. Phys.* **2021**, *155*, 104109.

[23] A. Schäfer, C. Huber, R. Ahlrichs, *J. Chem. Phys.* **1994**, *100*, 5829-5835.

[24] F. Neese, *WIREs Comput. Mol. Sci.* **2022**, *12*, e1606.

[25] J. C. Kromann, GitHub.

[26] W. Kabsch, *Acta. Cryst.* **1976**, *32*, 922-923.

[27] A. Šali, T. L. Blundell, *J. Mol. Biol.* **1993**, *234*, 779-815.

[28] C. Tian, K. Kasavajhala, K. A. A. Belfon, L. Raguette, H. Huang, A. N. Migues, J. Bickel, Y. Wang, J. Pincay, Q. Wu, C. Simmerling, *J. Chem. Theory Comput.* **2020**, *16*, 528-552.

[29] W. L. Jorgensen, J. Chandrasekhar, J. D. Madura, R. W. Impey, M. L. Klein, *J. Chem. Phys.* **1983**, *79*, 926-935.

[30] T. Lu, F. Chen, *J. Comput. Chem.* **2012**, *33*, 580-592.

[31] D. B. Langley, P. Schofield, D. Nevoltris, J. Jackson, K. J. L. Jackson, T. J. Peters, M. Burk, J. M. Matthews, A. Basten, C. C. Goodnow, S. van Nunen, J. H. Reed, D. Christ, *Proc. Natl. Acad. Sci. USA* **2022**, *119*, e2123212119.

[32] A. D. Becke, *Phys. Rev. A* **1988**, *38*, 3098-3100.

[33] C. Lee, W. Yang, R. G. Parr, *Phys. Rev. B* **1988**, *37*, 785-789.

[34] A. D. Becke, *J. Chem. Phys.* **1993**, *98*, 5648-5652.

[35] C. Riplinger, F. Neese, *J. Chem. Phys.* **2013**, *138*, 034106.

[36] C. Riplinger, B. Sandhoefer, A. Hansen, F. Neese, *J. Chem. Phys.* **2013**, *139*, 134101.

[37] C. Riplinger, P. Pinski, U. Becker, E. F. Valeev, F. Neese, *J. Chem. Phys.* **2016**, *144*, 024109.

[38] M. Saitow, U. Becker, C. Riplinger, E. F. Valeev, F. Neese, *J. Chem. Phys.* **2017**, *146*, 164105.

[39] Y. Guo, C. Riplinger, U. Becker, D. G. Liakos, Y. Minenkov, L. Cavallo, F. Neese, *J. Chem. Phys.* **2018**, *148*, 011101.

[40] W. B. Schneider, G. Bistoni, M. Sparta, M. Saitow, C. Riplinger, A. A. Auer, F. Neese, *J. Chem. Theory Comput.* **2016**, *12*, 4778-4792.

[41] J. Pipek, P. G. Mezey, *J. Chem. Phys.* **1989**, *90*, 4916-4926.

[42] R. A. Boto, F. Peccati, R. Laplaza, C. Quan, A. Carbone, J.-P. Piquemal, Y. Maday, J. Contreras-García, *J. Chem. Theory Comput.* **2020**, *16*, 4150-4158.

[43] W. Humphrey, A. Dalke, K. Schulten, *J Mol Graph.* **1996**, *14*, 33-38.

[44] S. Emamian, T. Lu, H. Kruse, H. Emamian, *J. Comput. Chem.* **2019**, *40*, 2868-2881.
